# Multi‐kinase framework promotes proliferation and invasion of lung adenocarcinoma through activation of dynamin‐related protein 1

**DOI:** 10.1002/1878-0261.12843

**Published:** 2020-12-11

**Authors:** Kuei‐Pin Chung, Yen‐Lin Huang, Yi‐Jung Chen, Yi‐Hsiu Juan, Chia‐Lang Hsu, Kiichi Nakahira, Yen‐Tsung Huang, Mong‐Wei Lin, Shang‐Gin Wu, Jin‐Yuan Shih, Yih‐Leong Chang, Chong‐Jen Yu

**Affiliations:** ^1^ Department of Laboratory Medicine National Taiwan University Hospital and National Taiwan University Cancer Center Taipei Taiwan; ^2^ Department of Pathology National Taiwan University Hospital and National Taiwan University Cancer Center Taipei Taiwan; ^3^ Department of Internal Medicine National Taiwan University Hospital Taipei Taiwan; ^4^ Department of Medical Research National Taiwan University Hospital Taipei Taiwan; ^5^ Department of Pharmacology Nara Medical University Kashihara, Nara Japan; ^6^ Institute of Statistical Science Academia Sinica Taipei Taiwan; ^7^ Department of Surgery National Taiwan University Hospital Taipei Taiwan; ^8^ Department of Internal Medicine College of Medicine National Taiwan University Taipei Taiwan; ^9^ Department and Graduate Institute of Pathology College of Medicine National Taiwan University Taipei Taiwan; ^10^ Department of Internal Medicine National Taiwan University Hospital Biomedical Park Hospital Zhubei City Taiwan

**Keywords:** cyclin‐dependent kinase 2, dynamin‐related protein 1, glycolytic serine synthesis, lung adenocarcinoma, mitochondria, prognosis

## Abstract

Recent studies revealed the role of dynamin‐related protein 1 (DRP1), encoded by the *DNM1L* gene, in regulating the growth of cancer cells of various origins. However, the regulation, function, and clinical significance of DRP1 remain undetermined in lung adenocarcinoma. Our study shows that the expression and activation of DRP1 are significantly correlated with proliferation and disease extent, as well as an increased risk of postoperative recurrence in stage I to stage IIIA lung adenocarcinoma. Loss of DRP1 in lung adenocarcinoma cell lines leads to an altered mitochondrial morphology, fewer copies of mitochondrial DNA, decreased respiratory complexes, and impaired oxidative phosphorylation. Additionally, the proliferation and invasion are both suppressed in DRP1‐depleted lung adenocarcinoma cell lines. Our data further revealed that DRP1 activation through serine 616 phosphorylation is regulated by ERK/AKT and CDK2 in lung adenocarcinoma cell lines. Collectively, we propose the multikinase framework in activating DRP1 in lung adenocarcinoma to promote the malignant properties. Biomarkers related to mitochondrial reprogramming, such as DRP1, can be used to evaluate the risk of postoperative recurrence in early‐stage lung adenocarcinoma.

## Graphical abstract

Here, we demonstrated that the multikinase framework including ERK/AKT and CDK2 promotes the proliferation and invasion of lung adenocarcinoma cell lines through activating DRP1. Our results further uncovered the prognostic significance of DRP1 in early‐stage lung adenocarcinoma, showing that the expression and activation of DRP1 are both significantly associated with an increased risk of postoperative recurrence.

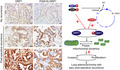

**Abbreviations**
AKT: protein kinase B
*ALK*: anaplastic lymphoma kinase
AMPK: 5′‐adenosine monophosphate‐activated protein kinase
ATP: adenosine triphosphate
ATF4: activating transcription factor
CRISPR: clustered regularly interspaced short palindromic repeats
Cas9: CRISPR‐associated protein 9
CDK: cyclin‐dependent kinase
DNA: deoxyribonucleic acid
DRP1: dynamin‐related protein 1
*DNM1L*: dynamin 1‐like
ECAR: extracellular acidification rate
EGFR: epidermal growth factor receptor
EMT: epithelial‐to‐mesenchymal transformation
ERK: extracellular signal‐regulated kinase
GTPase: guanosine triphosphatases
H‐score: histological score
IHC: immunohistochemistry
KO: knockout
MFN1: mitofusin 1
MFN2: mitofusin 2
mtDNA: mitochondrial DNA
mRNA: messenger RNA
NRF2: nuclear factor erythroid 2‐related factor 2
NTUH: National Taiwan University Hospital
OPA1: optic atrophy 1
OS: overall survival
OXPHOS: oxidative phosphorylation
PET: polyester
PFS: progression‐free survival
*PCK2*: phosphoenolpyruvate carboxykinase 2
*PHGDH*: phosphoglycerate dehydrogenase
*PSAT1*: phosphoserine aminotransferase 1
*PSPH*: phosphoserine phosphatase
P(S616)‐DRP1: phosphorylated DRP1 at serine 616
qPCR: quantitative polymerase chain reaction
RNA: ribonucleic acid
TCGA‐LUAD: the Cancer Genome Atlas Lung Adenocarcinoma
siRNA: small‐interfering RNA

## 1 Introduction

Mitochondrial dynamics, consisting of morphological changes associated with mitochondrial fission and fusion, are fundamental for metabolic reprogramming in response to various environmental stimuli [1]. Dynamin‐related protein 1 (DRP1), encoded by the *DNM1L* gene, regulates mitochondrial fission through interactions with various adaptors at the outer mitochondrial membrane [2]. Mitochondrial fission is required for precise mitophagy control [3, 4] and metabolic adaptations to energy stress [5]. *DNM1L* mutations are related to various neurologic diseases in humans [1], and loss of DRP1‐mediated mitochondrial fission is associated with cardiomyopathy [3], neurologic disorders [6], and macrophage dysfunction [7] in murine models.

Considering the role of mitochondrial dynamics in orchestrating cell metabolism, the cancer‐specific regulation of mitochondrial dynamics might be required to support proliferation, invasion, and metastasis [8, 9]. Studies have revealed that DRP1 is associated with tumor growth and invasion in pancreatic cancer [10, 11], breast cancer [12], and hepatocellular carcinoma [13], and cancer‐specific mitochondrial dynamics were suggested to be a potential therapeutic target [10, 11]. Studies have uncovered mitochondrial roles in the pathogenesis of human lung diseases [14, 15, 16], and upregulated DRP1 and mitophagy in the respiratory epithelium are implicated in the pathogenesis of chronic obstructive pulmonary disease [17]. However, the functional and clinical importance of DRP1 in lung adenocarcinoma remains elusive, and some previous studies were based on the effect of chemical mdivi‐1 [18, 19], which ineffectively inhibits the GTPase activity of human DRP1 [20]. Meanwhile, the clinical significance of DRP1 in lung adenocarcinoma is inconsistent among studies [21, 22, 23], which requires further exploration.

In this study, we aimed to clarify the function, regulation, and biological importance of DRP1 in lung adenocarcinoma, and the clinical significance of DRP1 expression and activation was extensively explored.

## 2 Materials and methods

### 2.1 Study population

The study protocol regarding human samples was approved by the Institutional Review Board at National Taiwan University Hospital (NTUH; 201807102RIND and 201911022RINC) and conformed to the standards set by the Declaration of Helsinki. Consecutive adult patients with lung adenocarcinoma diagnosed from January through December 2013 were identified using the database of the Cancer Registry, Medical Information Management Office of NTUH. The disease staging was evaluated according to the 7th edition of the American Joint Committee on Cancer. The study population consisted of patients who received definitive surgical treatment for stage I to stage IIIA lung adenocarcinoma. Patients were excluded if they had combined tumor histology, such as adenosquamous carcinoma. The written consent was obtained from each subject before the operation. We followed the study population to evaluate postoperative recurrence until 5 years after the surgery, and recurrence‐free survival was determined from the operation date until the first objective sign of recurrence.

### 2.2 Immunohistochemistry staining

Formalin‐fixed paraffin‐embedded tissue sections in 4 μm thickness were used for Immunohistochemistry (IHC) staining. The tissue sections were deparaffinized with xylene and then rehydrated with graded ethanol. The staining was performed using a Leica Biosystems BOND‐MAX autostainer or a Roche VENTENA BenchMark autostainer. The information on primary antibodies is summarized in Table S1. Antigen retrieval was performed using citrate buffer (pH 6.0), and the sections were subjected to sequential incubation with an endogenous peroxidase block, primary antibody, secondary antibody, diaminobenzidine, and hematoxylin. The IHC staining results of all tissue sections were evaluated randomly and blindly by a thoracic pathologist. Ki67 positivity was considered in cancer cells with nuclear staining of moderate intensity or higher. The positivity of DRP1 or phosphorylated DRP1 at serine 616 [P(S616)‐DRP1] was considered in cells with granular‐to‐diffuse cytoplasmic staining, and the intensity levels were graded from 0 to 3. The histological scores (H‐scores) of DRP1 and P(S616)‐DRP1 were calculated based on the following equation:

$$H‐\text{score}\;\left(0∼300\right)=\sum \left[\%cellsintensitylevel\left(0∼3\right)\right].$$

### 2.3 The Cancer Genome Atlas Lung Adenocarcinoma data analysis

The Cancer Genome Atlas Lung Adenocarcinoma (TCGA‐LUAD) clinical information and gene expression profiles were generated by the TCGA Research Network (https://www.cancer.gov/tcga) and were obtained using the R package TCGAbiolinks. To evaluate the association between *DNM1L* expression and prognosis, including progression‐free survival (PFS) and overall survival (OS), we categorized the tumors based on the median and the tertiles of *DNM1L* expression. The analyses were performed in the R environment (v3.6.1).

### 2.4 Cell culture, cell cycle synchronization, and reagents

Human lung adenocarcinoma cell line CL1‐0 was established as previously described [24, 25]. Lung adenocarcinoma cell lines, including A549 (CCL‐185), HCC827 (CRL‐2868), and H1975 (CRL‐5908), were purchased from the American Type Culture Collection (Manassas, VA, USA), and the PC9 cell line was a gift from Dr. James Chih‐Hsin Yang (Graduate Institute of Oncology, Cancer Research Center, National Taiwan University) [26]. All the cell lines were confirmed by cell line authentication performed by Genelabs Life Science Corporation (Taipei, Taiwan), and tested free of *Mycoplasma* infection. The cells were maintained in Roswell Park Memorial Institute (RPMI) 1640 medium (Gibco, Waltham, MA, USA) containing 10% fetal bovine serum (Biological Industries, Cromwell, CT) and 1% penicillin/streptomycin (Corning, Corning, NY, USA). CRISPR/Cas9 *DNM1L* and *CDK2* knockout (KO) cell lines were generated through lentiviral transduction, using the lentiCRISPRv2 vector (GenScript, Piscataway, NJ, USA) [27]. The sequences of guide RNA are described in Table S2. Sanger sequencing and immunoblotting were used to confirm knockout status. Transduction of the lentiCRISPRv2 vector was performed to generate control cells. For mitophagy evaluation, transduction of retroviral construct pCHAC‐mt‐mKeima, gift from Richard J. Youle (National Institute of Health) (Addgene plasmid #72342) [28, 29], was performed to express mtKeima in lung adenocarcinoma cell lines. Small‐interfering RNA (siRNA) transfection was performed as previously published [14], and the reagents are summarized in Table S1. Based on a published protocol [30], double thymidine block was used to synchronize cells at the G1/S phase, whereas sequential thymidine and nocodazole treatment was performed to synchronize cells at the G2/M phase. All chemical reagents used in this study are summarized in Table S1.

### 2.5 Colony formation and invasion assays

Colony formation assay was performed using a 6‐well culture plate, and 500 cells were added to each well. After culture for 10 or 14 days, the cells were fixed and stained with crystal violet. The area fraction in each well covered by the cells was measured using microscopic images and FIJI running imagej software (version 1.52b; https://fiji.sc/). Invasion assay was performed using a 24‐well plate and cell culture inserts with 8.0‐µm transparent polyester membrane (Corning, 353097). The Matrigel (Corning, 354234) was diluted to 2 mg/mL using RPMI medium and was used to coat the cell culture inserts. Cells and serum‐free RPMI medium were added to the cell culture insert and complete RPMI medium to the outer chamber. After culture for 16 h, the invading cells on the membrane were fixed and stained using Hoechst 33342. We obtained 8 images from each cell insert using the 10X objective, and the cell number was counted using FIJI running imagej software (version 1.51).

### 2.6 Mouse line and the xenograft model

All animal experiments were approved by the Institutional Animal Care and Use Committee at Medical College, National Taiwan University Hospital. Nude mice were purchased from the National Laboratory of Animal Center. Male nude mice at 6 weeks of age were used for control or *DNM1L*‐KO CL1‐0 xenograft implantation [31]. For each mouse, 5 × 10^6^ cells were prepared in serum‐free RPMI1640 medium and mixed with Matrigel (Corning) in 1:1 ratio on ice. The cell suspension with a final volume of 100 μL was injected subcutaneously at the flank. The tumor volume was recorded weekly after implantation and was calculated using the following equation: tumor volume (mm^3^) = length (mm) × (width (mm))^2^/2. The mice were euthanized 5 weeks after tumor implantation, and the tumor weight was measured after dissection.

### 2.7 Immunoblotting

Protein extraction was performed as previously published [14], and proteins were resolved with 8–12% Tris/Glycine gels. The primary and secondary antibodies for immunoblotting are summarized in Table S1. Densitometric quantification of bands was performed using FIJI running ImageJ software.

### 2.8 Confocal microscopy

Mitochondrial and nuclear staining was performed as previously published [14], and the reagents are summarized in Table S1. The images were obtained using a Zeiss LSM 880 Laser Scanning Microscope, and a 3 × 3 tile images acquired using 63X/1.4 objective formed a high‐power field. Three high‐power fields per dish were used for mitochondrial quantification.

### 2.9 Extracellular flux analysis

The oxygen consumption rate and extracellular acidification rate (ECAR) were measured with a Seahorse XFe24 Analyzer (Agilent, Santa Clara, CA). The assays were performed using the Mito Stress Test Kit (Agilent, 103015‐100), based on the manufacturer’s instructions. The data were normalized using the CyQUANT Cell Proliferation Assay Kit (Invitrogen, C7026, Waltham, MA) and analyzed using Wave software (version 2.6.1.38).

### 2.10 Real‐time quantitative polymerase chain reaction (qPCR)

Real‐time qPCR was applied to measure mRNA expression and mitochondrial DNA (mtDNA) quantification. The primer sequences for qPCR are summarized in Table S2. TRIzol Reagent (Invitrogen, 15596026) and TriRNA Pure Kit (Geneaid TRP100, New Taipei, Taiwan) were used for RNA extraction. Reverse transcription was performed using the High‐Capacity cDNA Reverse Transcription Kit (Applied Biosystems, 4368813, Waltham, MA). The DNeasy Blood and Tissue Kit (Qiagen, 69506, Hilden, Germany) was used for DNA extraction for mitochondrial DNA (mtDNA) quantification. Power SYBR Green PCR Master Mix (Applied Biosystems, 4367659) was used for qPCRs in an Applied Biosystems 7900HT Fast Real‐Time PCR System. The 2^−ΔΔCt^ method was used to calculate RNA expression relative to *TBP*, and mtDNA copy number relative to genomic DNA copy number.

### 2.11 Flow cytometric analysis of the cell cycle

A FACSCalibur flow cytometer (BD Biosciences, San Jose, CA) was used for cell cycle analysis. The cells were stained using the Propidium Iodide Flow Cytometry Kit (ab139418, Abcam, Cambridge, MA), based on the manufacturer’s instructions. Mitophagy detection was performed on a FACSAria III (BD Biosciences) in the Flow Cytometric Analyzing and Sorting Core Facility at NTUH, using the cells expressing pH‐sensitive mtKeima fluorescence. Excitation using 405‐nm and 561‐nm laser was used to detect mtKeima at pH 7.0 and at pH 4.0, respectively [14]. Identical gating configuration, including upper and lower gates (see also Fig. S4), was used for evaluating mitophagy in all the cell lines, and the percentages of the cells in the upper gate were used for mitophagy intensity quantification. All the flow cytometric data were analyzed using FlowJo software (version 10.0, BD Biosciences).

### 2.12 Statistical analysis

For differences in continuous variables, Student’s t‐test or Mann–Whitney U‐test was used for comparisons between two groups as appropriate, whereas one‐way ANOVA with a Bonferroni post hoc test was used for multigroup comparisons. Categorical variables were compared by Pearson’s chi‐square test or Fisher’s exact test, as appropriate. The dichotomization of the study population was performed based on the H‐score median or the optimized H‐score cutoff determined by receiver operating characteristic curves and the Youden index. For evaluating the correlation between two continuous variables, the correlation coefficient was calculated using both the linear regression analysis and the Spearman correlation analysis. Kaplan–Meier curves were plotted for recurrence‐free survival, progression‐free survival, or overall survival, and survival differences were compared using log‐rank tests. Multivariate Cox proportional hazard models were used to calculate the hazard ratio (HR) for postoperative recurrence. A two‐sided *p* value less than 0.05 was considered statistically significant. All analyses were performed using SPSS (version 17.0; IBM Corporation, Armonk, NY) or GraphPad Prism (version 5.0; GraphPad Software, La Jolla, CA).

## 3 Results

### 3.1 DRP1 is significantly associated with early postoperative recurrence in lung adenocarcinoma

**Fig. 1:**
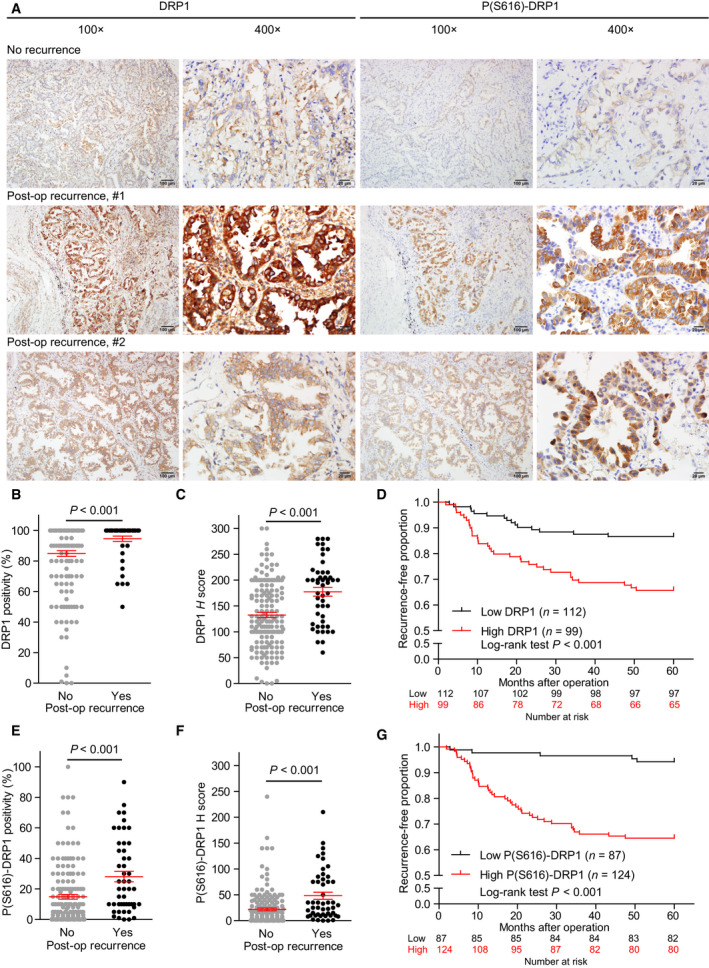
DRP1 activation and expression are significantly associated with the postoperative recurrence of lung adenocarcinoma. (A) Representative immunohistochemistry (IHC) staining of DRP1 and phosphorylated DRP1 using sections from paraffin‐embedded lung adenocarcinoma samples. The images were obtained under 100×or 400×magnification. (B and C) Association between postoperative recurrence and DRP1 expression, quantified by positivity and H‐score, in lung adenocarcinoma, based on IHC staining. (D), Kaplan–Meier curves to evaluate DRP1 H‐score and recurrence‐free survival, calculated from the date of surgery. The study population was dichotomized based on the H‐score of DRP1. (E and F) Association between postoperative recurrence and DRP1 activation, quantified by positivity and H‐score of phosphorylated DRP1, in lung adenocarcinoma, based on IHC staining. (G) Kaplan–Meier curves to evaluate the H‐score of phosphorylated DRP1 and recurrence‐free survival. The study population was dichotomized based on the H‐score of phosphorylated DRP1. The cutoff for dichotomization in (D) and (G) was optimized and determined by receiver operating characteristic curves and the Youden index. Data in B, C, E, and F are presented as the mean ± standard error, and differences were compared using Student’s t‐test. The differences in survival were compared using a log‐rank test.

To explore the prognostic significance of DRP1 in lung adenocarcinoma, we first analyzed TCGA‐LUAD data. We found that tumors with *DNM1L* expression above the median were significantly associated with worse PFS (Fig. S1A), and a nonsignificant trend of worse OS (Fig. S1B). The analysis further revealed that tumors with *DNM1L* expression above the 65th percentile, compared to tumors with *DNM1L* expression less than the 35th percentile, were significantly associated with decreased PFS and OS (Fig. S1C–D). The above results thus suggested that DRP1 expression may be a prognostic biomarker in lung adenocarcinoma. Since the survival of patients with advanced lung adenocarcinoma may be affected by various anticancer treatments, we explored the clinical significance of DRP1 specifically in patients with operable lung adenocarcinoma. Two hundred and eleven patients receiving definitive surgical treatment for lung adenocarcinoma during the study period were enrolled. The clinical characteristics are summarized in Table S3. Whereas DRP1 staining was sometimes present in the normal tissues adjacent to cancer cells, P(S616)‐DRP1 staining was exclusively positive in cancer cells (Fig. 1A). We also found that the H‐score of DRP1 in lung adenocarcinoma was significantly correlated with that of P(S616)‐DRP1 (Fig. S2A). This finding suggested that upregulated DRP1 expression is accompanied by simultaneous DRP1 activation in lung adenocarcinoma. Compared to that in patients without postoperative recurrence, those with recurrence had lung adenocarcinoma with increased expression of both DRP1 (Fig. 1B,C) and P(S616)‐DRP1 (Fig. 1E,F). Further, we found that DRP1 expression in adjacent noncancerous tissues was not significantly associated with postoperative recurrence in patients with lung adenocarcinoma (Fig. S2B). Meanwhile, 131 patients (62.1%) in the study population had stage I lung adenocarcinoma, and our results further revealed that DRP1 and P(S616)‐DRP1 expression remained significantly associated with postoperative recurrence in patients with early‐stage lung adenocarcinoma (Fig. S2C–F). We then dichotomized the study population based on DRP1 or P(S616)‐DRP1 H‐score, and the cutoff for dichotomization was determined by the median (Fig. S3) or the Youden index (Fig. 1D, G). The results showed that lung adenocarcinoma with a high DRP1 or P(S616)‐DRP1 H‐score was significantly associated with early postoperative recurrence. We conducted multivariate regression analyses, and the results showed that lung adenocarcinoma with a high DRP1 (HR = 2.451, 95% confidence interval [CI] = 1.266–4.744, *p* = 0.008) or P(S616)‐DRP1 (HR = 5.261, 95% CI = 2.008–13.785, *p* = 0.001) H‐score was independently significantly associated with 5‐year postoperative recurrence (Table S4).

### 3.2 DRP1 regulates mitochondrial morphology and biogenesis in lung adenocarcinoma cell lines

**Fig. 2:**
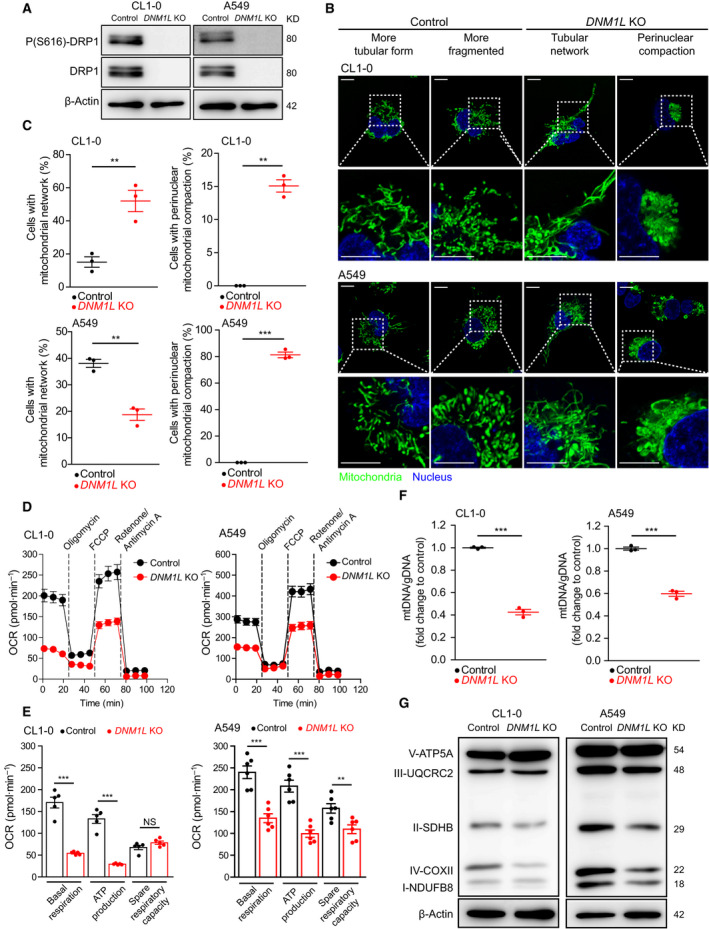
Loss of DRP1 leads to altered mitochondrial morphology, impaired mitochondrial respiration, and decreased mitochondrial DNA (mtDNA) copies and respiratory complexes. (A) Immunoblots showing the *DNM1L* CRISPR/Cas9 knockout (KO) efficacy in CL1‐0 and A549 cell (*n* = 2 technical repeats). (B) Live‐cell confocal imaging of mitochondrial morphology in control and *DNM1L*‐KO lung adenocarcinoma cell lines (*n* = 3 technical repeats, scale bar = 10 μm). The mitochondria were stained using MitoTracker Green, and nuclei were labeled using Hoechst 33342. (C) Quantification of the mitochondrial morphology using confocal imaging. Each dot indicates a glass‐bottom dish, and three high‐power fields, consisting of 3 × 3 tile images obtained using the 63X/1.4 objective per dish, were used for quantification. Each high‐power field contains more than 100 cells. (D) Extracellular flux analysis to measure oxygen consumption rate (OCR) in control and *DNM1L*‐KO lung adenocarcinoma cells (*n* = 2 technical repeats). Results of each cell line comprised five (CL1‐0) or six replicates (A549). (E) Summary of the extracellular flux analysis data. (F) Quantification of mitochondrial DNA (mtDNA) copy numbers, which were measured using real‐time quantitative PCR and calculated relative to genomic DNA (gDNA; *n* = 2 technical repeats). (G) Immunoblots for mitochondrial respiratory complex I to complex V (*n* = 2 technical repeats). Data in C, E, and F are presented as the mean ± standard error, and the differences were compared using Student’s t‐test (NS, nonsignificant, ** *P* < 0.01, *** *P* < 0.001).

Our clinical data suggested that the expression and activation of DRP1 may regulate the malignant features of lung adenocarcinoma. To investigate the potential mechanisms, we first investigated the biological role of DRP1 in lung adenocarcinoma cell lines and generated *DNM1L*‐KO CL1‐0 and A549 cells (Fig. 2A). Since DRP1 is essential for regulating mitochondrial fission [6], we examined changes in mitochondrial morphology in *DNM1L*‐KO cells. We found that the mitochondria could be a tubular or fragmented shape in both CL1‐0 and A549 cells (Fig. 2B). In CL1‐0 cells, depleting DRP1 led to the formation of a mitochondrial tubular network in most cells, whereas perinuclear mitochondrial compaction occurred in some cells. In contrast, most *DNM1L*‐KO A549 cells showed perinuclear mitochondrial compaction (Fig. 2B,C). Perinuclear compaction of mitochondria is associated with mitophagy activation [17], and the mitochondrial morphology in *DNM1L*‐KO A549 cells may suggest an augmented mitophagy response to mitochondrial damage. We further investigated the mitophagy activity at the basal state and after mitochondrial injury by oligomycin and antimycin A, using mtKeima‐expressing CL1‐0 and A549 cells (Fig. S4A–B, D–E) [14]. Our results demonstrated that DRP1 depletion modestly increased mitophagy activity in CL1‐0 and A549 at the basal states (Fig. S4C). Furthermore, we found that, when mitochondria were injured, the mitophagy activity was more enhanced in *DNM1L*‐KO cells than in the control (Fig. S4F) and was more intense in A549 cells than in CL1‐0 cells (Fig. S4G). Therefore, in consistence with mitochondrial morphological findings, our results indicated that DRP1 depletion in different lung adenocarcinoma cell lines variably augmented mitophagy at baseline and in responses to mitochondrial damage.

Proper mitochondrial dynamics are crucial for mitochondrial bioenergetics [3, 32], and DRP1 depletion resulted in profoundly impaired mitochondrial respiration in lung adenocarcinoma cell lines (Fig. 2D), leading to significantly decreased mitochondrial ATP production (Fig. 2E). To evaluate the mechanisms underlying the decrease in mitochondrial respiration, we examined the mtDNA copy number and respiratory complex in *DNM1L*‐KO lung adenocarcinoma cell lines. The results showed that DRP1 depletion in lung adenocarcinoma cell lines caused a significant decrease in mtDNA copy numbers (Fig. 2F) and selectively decreased the levels of respiratory complexes I, II, and IV (Fig. 2G). These data indicated that DRP1 loss perturbs mitochondrial dynamics in lung adenocarcinoma cell lines and suppresses oxidative phosphorylation (OXPHOS), partly due to impaired mtDNA biogenesis and the downregulation of respiratory complexes.

### 3.3 DRP1 expression is required to promote proliferation and invasion of lung adenocarcinoma cell lines

**Fig. 3:**
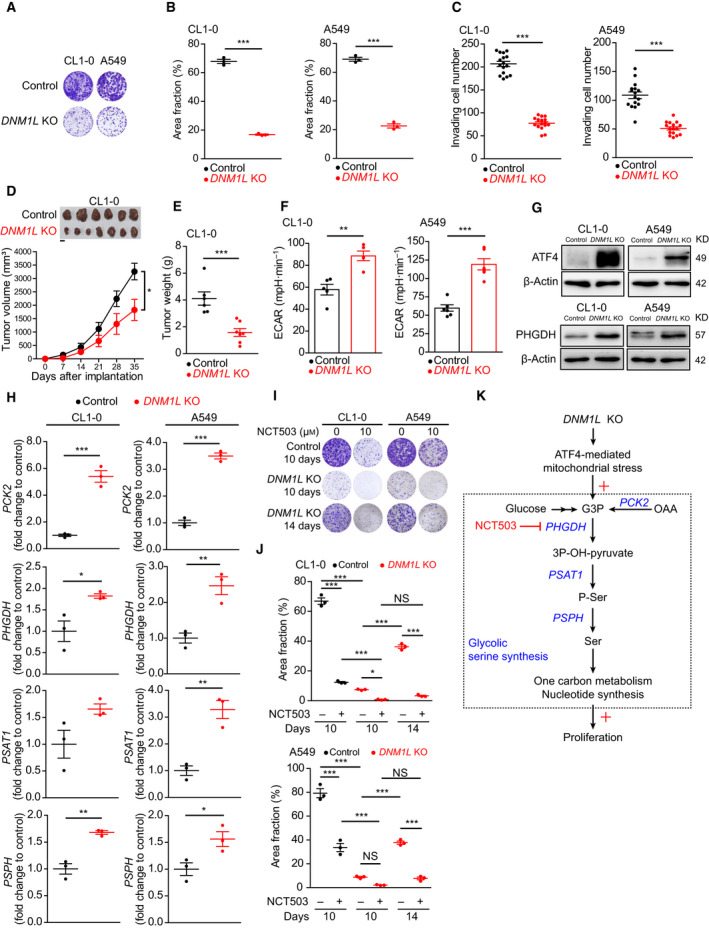
Loss of DRP1 inhibits proliferation and invasiveness and induces glycolytic serine synthesis in lung adenocarcinoma. (A) The representative well images of the colony formation assay. (B) Quantification of the colony formation assay. Each dot indicates a well in a 6‐well culture plate (*n* = 2 technical repeats). (C) Quantification of invasion assays based on counting invading cell numbers. Each dot indicates a fluorescent microscopy field under a 10 × objective (*n* = 4 technical repeats). (D, E) Xenograft implantations of control (*n* = 6 mice) and *DNM1L*‐KO (*n* = 7 mice) CL1‐0 cells showed that the depletion of DRP1 inhibits *in vivo* tumor growth (D, scale bar = 1 cm), leading to significantly decreased tumor volume (D) and tumor weight (E). (F) Extracellular acidification rate (ECAR) measured using a Seahorse XFe24 analyzer. For each cell, five (CL1‐0) or six (A549) replicates were measured (*n* = 2 technical repeats). (G) Immunoblots showing increased ATF4 and PHGDH protein expression in CL1‐0 and A549 cells after *DNM1L* knockout (*n* = 2 technical repeats). (H) Real‐time quantitative PCRs to measure the levels of mRNA related to ATF4‐mediated mitochondrial stress. For each cell line, three replicates were measured (*n* = 2 technical repeats). (I) Representative well images of colony formation assay after treatment with solvent or NCT503. (J) Quantification of the colony formation assay. Each dot indicates a well in a 6‐well culture plate (*n* = 2 technical repeats). (K) Scheme showing that the loss of DRP1 induces ATF4‐mediated mitochondrial stress responses, characterized by glycolytic serine synthesis and the downstream one‐carbon metabolism and nucleotide synthesis. Data in B, C, D, E, F, H, and J are presented as the mean ± standard error. The differences were compared using Student’s *t*‐test in B, C, D, E, F, and H, and by one‐way ANOVA with a Bonferroni post hoc test in J (NS, nonsignificant, *P < 0.05, **P < 0.01, ***P < 0.001).

Proper regulation of mitochondrial dynamics is required for cells to adapt to various environmental stress [33], and it is assumed to be essential for the malignant properties of cancer cells [9]. Our results revealed that the *in vitro* colony formation (Fig. 3A–B) and invasiveness (Fig. 3C), and the *in vivo* xenograft tumor growth (Fig. 3D,E) were all inhibited in lung adenocarcinoma cell lines after DRP1 depletion. We subsequently evaluated whether the expression and activation of DRP1 in surgical samples of lung adenocarcinoma were associated with proliferation and disease extent. We found that the intensity of P(S616)‐DRP1 correlated with Ki67 positivity (Fig. S5A). In addition, we found that the H‐scores of DRP1 and P(S616)‐DRP1 were significantly correlated with Ki67 positivity (Fig. S5B–C) and were significantly increased in stage IB–stage IIIA lung adenocarcinoma (Fig. S5D–E). Therefore, our data from clinical samples supported our *in vitro* findings, suggesting that DRP1 expression is crucial for the aggressiveness of lung adenocarcinoma. Given that OXPHOS was suppressed in *DNM1L*‐KO lung adenocarcinoma cell lines, we next examined whether OXPHOS was required for the proliferation and invasion of lung adenocarcinoma cell lines. Our results showed that oligomycin, an inhibitor to ATP synthase, markedly inhibited the colony formation capabilities (Fig. S6A–B) but surprisingly enhanced the invasiveness (Fig. S6C) of lung adenocarcinoma cell lines. Therefore, although impaired OXPHOS contributes to the impaired proliferation in *DNM1L*‐KO lung adenocarcinoma cell lines, it cannot account for the anti‐invasive effects after DRP1 depletion.

### 3.4 Glycolytic serine synthesis is required for the proliferation of DRP1‐depleted lung adenocarcinoma cell lines

Our *in vivo* results (Fig. 3D) demonstrated that growth arrest was not induced in lung adenocarcinoma cell lines after depletion of DRP1, and the findings suggested that adaptive responses after DRP1 loss may support the proliferation of cancer cells. ATF4‐mediated mitochondrial stress responses are induced by mitochondrial damage and defective mitochondrial fusion [14, 34], and the hallmark is glycolytic serine synthesis, which is required for one‐carbon metabolism, nucleotide synthesis, redox homeostasis, and glycerophospholipid production [34, 35, 36, 37]. We thus investigated the role of mitochondrial stress responses in regulating proliferation of DRP1‐depleted lung adenocarcinoma cell lines. Our results revealed enhanced ECAR in *DNM1L*‐KO lung adenocarcinoma cell lines, indicating upregulated glycolysis (Fig. 3F). Additionally, we found increased protein expression of ATF4 and PHGDH (Fig. 3G), along with upregulated mRNA levels of *PCK2*, *PHGDH*, *PSAT1*, and *PSPH* (Fig. 3H), in *DNM1L*‐KO lung adenocarcinoma cell lines. These findings indicated that the ATF4‐mediated glycolytic serine synthesis pathway is activated in DRP1‐depleted lung adenocarcinoma cell lines. We further used NCT503 to block PHGDH [37], the rate‐limiting enzyme of glycolytic serine synthesis, and evaluated the role of this adaptive response in the proliferation of *DNM1L*‐KO lung adenocarcinoma cell lines. The results showed that inhibiting glycolytic serine synthesis remarkably suppressed the proliferation of *DNM1L*‐KO lung adenocarcinoma cell lines (Fig. 3I,J). These findings indicated that, while DRP1‐mediated mitochondrial fission is required to effectively promote the proliferation and invasion of lung adenocarcinoma cell lines, the mitochondrial stress response can support the proliferation of lung adenocarcinoma cell lines without activated mitochondrial dynamics (Fig. 3K).

### 3.5 ERK and AKT signaling pathways regulate phosphorylation of DRP1 at serine 616 in lung adenocarcinoma cell lines

**Fig. 4:**
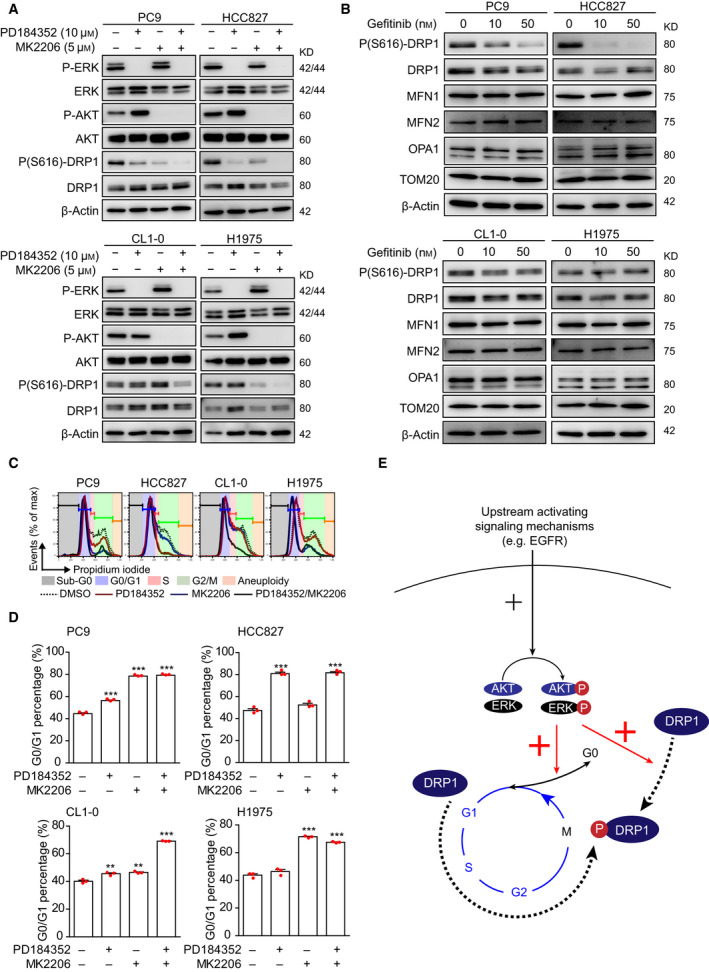
ERK and AKT regulate DRP1 phosphorylation at serine 616. (A) Immunoblots showing the effects of ERK inhibitor (PD184352) and AKT inhibitor (MK2206) on the regulation of DRP1 phosphorylation at serine 616 after treatment for 24 h (*n* = 3 technical repeats). (B) Immunoblots showing the effects of the EGFR tyrosine kinase inhibitor gefitinib on the expression of protein machinery that regulates mitochondrial dynamics after treatment for 24 h (*n* = 3 technical repeats). (C) Representative flow cytometric histograms showing cell cycle progression in response to ERK and AKT inhibition for 24 h in different lung adenocarcinoma cell lines (*n* = 2 technical repeats). (D) The percentages of cells in G0/G1 phases with ERK and AKT inhibitors. Data are presented as the mean ± standard error, and the differences were compared using Student’s *t*‐test (**P < 0.01, ***P < 0.001). (E) Schematic showing the hypothetical model of the direct and indirect regulation of DRP1 activation by ERK and AKT.

Our clinical data (Fig. S2A) suggested that DRP1 is maintained in the activated state in lung adenocarcinoma. However, the mechanism regulating DRP1 activation in lung adenocarcinoma is not clear. The phosphorylation of DRP1 at serine 616, which activates GTPase activity [38], is mediated by CDK1 in HeLa cells [39] and is regulated by ERK in pancreatic cancer cell lines [10]. Therefore, the regulation of DRP1 activity is cell‐specific. Since the proliferation and survival of lung adenocarcinoma are principally driven by ERK and AKT downstream signaling, we thus examined the effects of an ERK inhibitor (PD184352) and AKT inhibitor (MK2206) on DRP1 phosphorylation, and the findings revealed that the roles of ERK and AKT in DRP1 activation varied in different lung adenocarcinoma cells (Fig. 4A). In PC9 and HCC827 cells, both ERK and AKT regulated DRP1 phosphorylation. However, AKT but not ERK was involved in DRP1 activation in H1975 cells, whereas the inhibition of both ERK and AKT was required to decrease P(S616)‐DRP1 in CL1‐0 cells. Several upstream signaling pathways can activate ERK and AKT in lung adenocarcinoma [40], and may regulate DRP1 phosphorylation through ERK and AKT. EGFR signaling is one important driving mechanism in lung adenocarcinoma [41], and we further examined whether EGFR regulates the activation of DRP1. We found that gefitinib deactivated both ERK and AKT in PC9 cells, inhibited ERK activation in HCC827 cells, but did not affect ERK or AKT activation in resistant CL1‐0 and H1975 cells (Fig. S7A). Consistently, gefitinib treatment decreased P(S616)‐DRP1 in PC9 and HCC827 cells, but not in resistant cell lines (Figs 4B and S7B). Meanwhile, gefitinib treatment did not alter the expression of DRP1 (Fig. S7B) or proteins related to mitochondrial fusion, including MFN1, MFN2, and OPA1. To further confirm that the EGFR downstream signaling pathway regulates DRP1 activation, we treated H1975 using osimertinib and stimulated CL1‐0 using EGF. Our results showed that osimertinib inhibited the activation of ERK and AKT in H1975 and decreased P(S616)‐DRP1 expression (Fig. S7C). Additionally, EGF treatment effectively activated ERK and AKT in CL1‐0 and increased P(S616)‐DRP1 (Fig. S7D). The data above together indicated that the upstream activating mechanisms, such as EGFR, can activate DRP1 in lung adenocarcinoma cell lines through ERK and AKT signaling.

### 3.6 DRP1 activation is maintained by a multikinase mechanism in lung adenocarcinoma cell lines

**Fig. 5:**
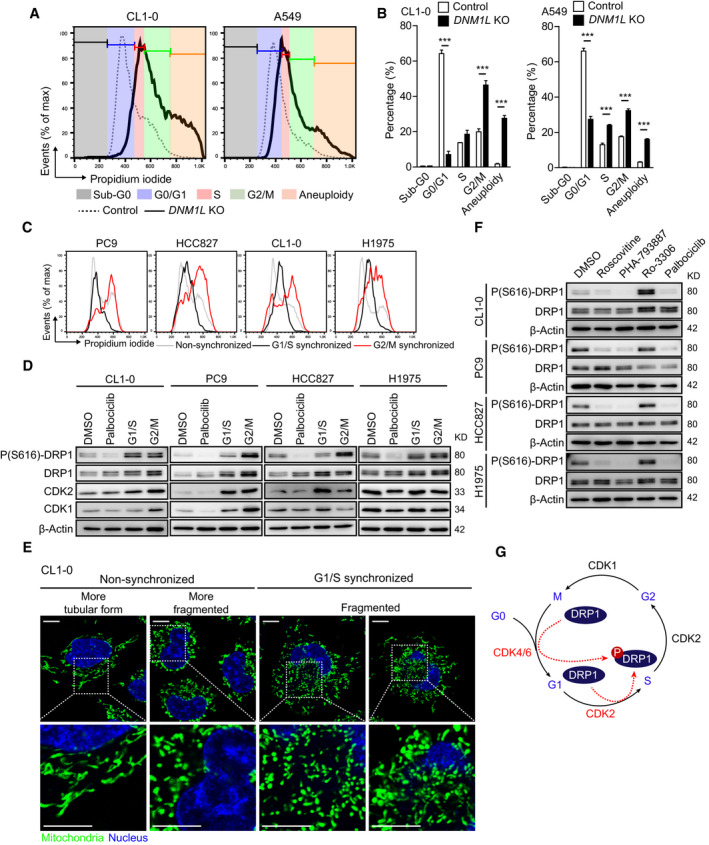
Entry into the cell cycle induces the phosphorylation of DRP1 at serine 616. (A) Representative flow cytometric histogram showing cell cycle progression of control and *DNM1L* knockout lung adenocarcinoma cells (*n* = 2 technical repeats). (B) Percentage of cells at different phases of the cell cycle based on flow cytometric analyses. Data are presented as the mean ± standard error, and the differences were compared using Student’s *t*‐test (***P < 0.001). (C) Representative flow cytometric analysis to confirm the efficacy of G1/S and G2/M cell cycle synchronization (*n* = 2 technical repeats). (D) Immunoblots to show the levels of phosphorylated DRP1 upon unsynchronized, palbociclib treatment for 24 h and in G1/S or G2/M‐synchronized states (*n* = 3 technical repeats). (E) Representative live‐cell confocal imaging of mitochondrial morphology in unsynchronized or G1/S‐synchronized H1605 cells (*n* = 2 technical repeats, scale bar = 10 μm). The mitochondria were stained using MitoTracker Green, and nuclei were labeled using Hoechst 33342. (F) Immunoblots to show the effects of CDK inhibitors on the regulation of DRP1 phosphorylation after treatment for 24 h (*n* = 3 technical repeats). (G) A hypothetical scheme showing how DRP1 is phosphorylated after entry into the cell cycle.

Given that DRP1 can be phosphorylated during cell cycle progression [24, 39], we evaluated whether ERK or AKT inhibitors could prevent cell cycle entry. Results showed that the degree of G0/G1 arrest with ERK and/or AKT inhibition resembled the decrease in P(S616)‐DRP1 (Fig. 4C,D). Therefore, ERK and AKT might activate DRP1 through direct regulation or indirectly by promoting cell cycle entry (Fig. 4E). To prove our hypothetical model, we first examined whether DRP1 depletion affected cell cycle progression in lung adenocarcinoma. Results showed that the CRISPR/Cas9 lentiviral transduction did not affect cell cycle progression in either CL1‐0 or A549 (Fig. S8), and further revealed an increased percentage of cells at S/G2/M phases in *DNM1L*‐KO lung adenocarcinoma cell lines (Fig. 5A,B), indicating that DRP1 activity is required for correct cell cycle progression. To evaluate DRP1 phosphorylation at different cell cycle phases, we used palbociclib to induce G0/G1 arrest (Fig. S9A,B) and synchronized cells at G1/S or G2/M phases (Fig. 5C). Results revealed that palbociclib treatment, but not G1/S or G2/M synchronization, significantly decreased DRP1 phosphorylation (Fig. 5D). In addition, we observed highly fragmented mitochondria in CL1‐0 cells synchronized at the G1/S phase (Fig. 5E), suggesting increased mitochondrial fission. Therefore, our data indicated that DRP1 was phosphorylated early after entry into the cell cycle. Since various CDKs regulate cell cycle entry and progression, we then evaluated the effects of various CDK inhibitors on DRP1 phosphorylation in lung adenocarcinoma cell lines. In addition to palbociclib, we found that both PHA‐793887 and roscovitine could decrease DRP1 phosphorylation (Fig. 5F). Based on the activity of these CDK inhibitors (Fig. S9A–C), we speculated that CDK2, CDK4/6, and CDK5 are candidates that activate DRP1 during the cell cycle in lung adenocarcinoma cell lines (Fig. 5G).

**Fig. 6:**
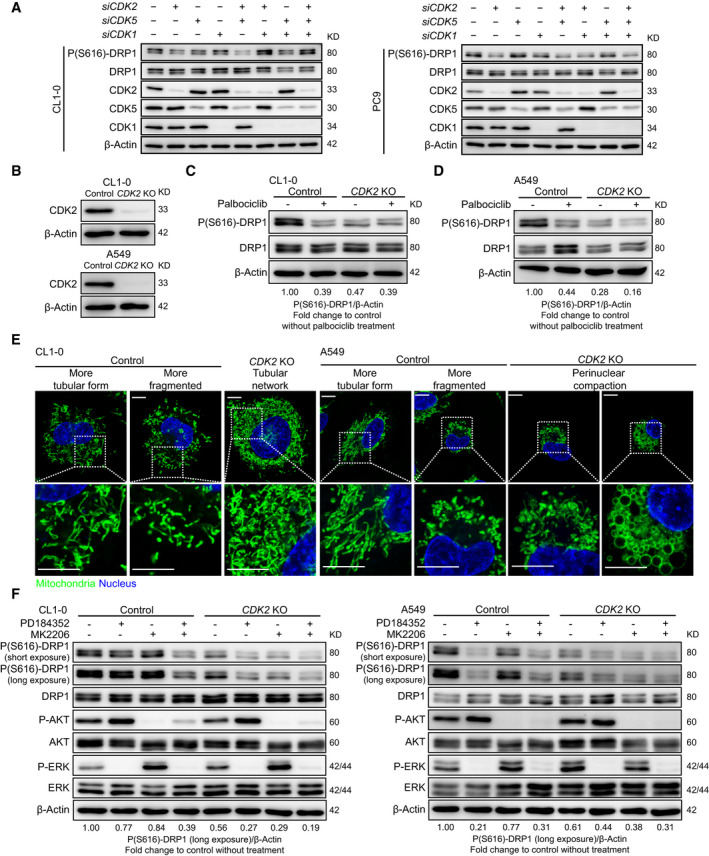
DRP1 is activated through a multikinase model in lung adenocarcinoma. (A) Immunoblots to evaluate the regulatory effects of CDK on DRP1 phosphorylation based on small‐interfering RNA knockdown of CDK1, CDK2, and CDK5 (*n* = 3 technical repeats). (B) Immunoblots to confirm the CDK2 CRISPR/Cas9 knockout (KO) efficacy (*n* = 2 technical repeats). (C and D) Immunoblots to evaluate the role of CDK4/6 in phosphorylating DRP1 using the CDK4/6 inhibitor palbociclib (treatment for 24 hours; *n* = 2 technical repeats). (E) Representative live‐cell confocal imaging of mitochondrial morphology in control and *CDK2*‐KO CL1‐0 or A549 cells (*n* = 3 technical repeats, scale bar = 10 μm). The mitochondria were stained using MitoTracker Green, and nuclei were labeled using Hoechst 33342. (F) Immunoblots to evaluate the role of ERK/AKT in directly regulating DRP1 phosphorylation using ERK and AKT inhibitors in control or *CDK2*‐KO lung adenocarcinoma cell lines (treatment for 24 h; *n* = 2 technical repeats).

To elucidate the regulatory effect of CDKs on DRP1 phosphorylation in lung adenocarcinoma cell lines, we applied siRNA transfection to knock down *CDK1*, *CDK2*, and *CDK5*. The data revealed that *CDK2* knockdown consistently decreased P(S616)‐DRP1 in CL1‐0 and PC9 cells (Fig. 6A and Fig. S10A). We then generated *CDK2*‐KO CL1‐0 and A549 cell lines (Fig. 6B) and found that P(S616)‐DRP1 was decreased after CDK2 depletion (Fig. 6C,D). To examine the role of CDK4/6 in activating DRP1, we evaluated whether palbociclib could decrease P(S616)‐DRP1 in *CDK2*‐KO cells. The results showed that it induced a very modest decrease in P(S616)‐DRP1 in *CDK2*‐KO CL1‐0 (Fig. 6C) and *CDK2*‐KO A549 (Fig. 6D) cells. Interestingly, depleting CDK2 in lung adenocarcinoma cell lines resulted in similar mitochondrial morphological changes caused by DRP1 depletion and led to an extensive mitochondrial tubular network in CL1‐0 cells and perinuclear compaction of swollen mitochondria in A549 cells (Fig. 6E and Fig. S10B). These data thus confirmed that CDK2 is the key regulator of DRP1 and mitochondrial fragmentation in lung adenocarcinoma cell lines during cell cycle progression.

To clarify the potential role of ERK and AKT in directly regulating DRP1 activation, we examined whether ERK and AKT inhibitors resulted in DRP1 dephosphorylation in *CDK2*‐KO cells. The results showed that both together decreased P(S616)‐DRP1 in *CDK2*‐KO CL1‐0 and A549 cells (Fig. 6F). Collectively, our results uncovered a multikinase scheme specific to lung adenocarcinoma cell lines that activates DRP1, and provided mechanistic insights suggesting that mitochondrial reprogramming through the upregulation of mitochondrial dynamics is a cornerstone of cancer development and is required for the proliferation and invasiveness of lung adenocarcinoma (Fig. S11).

## 4 Discussion

Here, we provide both experimental evidence and clinical evidence supporting the requirement of DRP1 for malignant features of lung adenocarcinoma. Our investigation helped to decipher the clinical significance of DRP1 in patients with early‐stage lung adenocarcinoma, showing that the increase in DRP1 expression and activation is correlated with enhanced proliferation and disease extent and is independently associated with early postoperative recurrence.

Although deleting *DNM1L* does not affect OXPOS and mtDNA synthesis in mouse embryonic fibroblasts [6], our results are similar to those of pancreatic cancers [11] and revealed that the loss of DRP1 in lung adenocarcinoma cell lines leads to increased mitophagy to mitochondrial damage and decreased OXPHOS and mtDNA copy numbers. Our data showed that the mitochondria are not in exactly the same shape in CL1‐0 and A549 after loss of DRP1. Previous studies showed that the mitochondrial morphology is determined by the machinery regulating mitochondrial dynamics [1], and the metabolic demands of the cells [42]. The different mitochondrial shape in CL1‐0 and A549 may thus reflect different metabolic states of the cells. The augmented mitophagy response to mitochondrial injury in A549 may also contribute to the mitochondrial morphological alternations after DRP1 depletion. Meanwhile, we also found that the blockade of OXPHOS promotes the invasiveness of lung adenocarcinoma cell lines. A recent study showed that OXPHOS impairment in lung adenocarcinoma cell lines induces AMPK‐mediated epithelial‐to‐mesenchymal transformation (EMT) and promotes invasion [43]. Meanwhile, it was shown recently that EMT can activate mitochondrial fusion to regulate the polarity of stem cells [44]. The findings that DRP1 depletion in lung adenocarcinoma cell lines suppresses OXPHOS without enhancing invasiveness might be due to the perturbation of mitochondrial dynamics. Given that EMT activation results in resistance to EGFR kinase inhibitors [26], studies are required to delineate mitochondrial and metabolic alterations required for EMT activation and to explore potential therapies to overcome EMT‐related resistance.

The proliferation, invasion, and metastasis of cancer cells require precise metabolic control [8], and the support of adequate biosynthetic and bioenergetic functions. Although impaired mitochondrial fusion has been shown to perturb lipid metabolic homeostasis [14], the detailed biosynthetic changes when DRP1‐mediated mitochondrial fission in lung adenocarcinoma cell lines is impaired are unknown. Altered substrate synthesis in lung adenocarcinoma cell lines after DRP1 depletion is suggested by the activation of mitochondrial stress responses. Furthermore, since DRP1 might not be a suitable therapeutic target due to the crucial role of mitochondrial dynamics in cellular adaptations [1], the biosynthetic alterations after DRP1 depletion might suggest potential metabolism‐related therapeutic targets in lung adenocarcinoma. The exact changes in substrate utilization and synthesis, which explain the biologic effects in DRP1‐depleted lung adenocarcinoma cell lines, thus should be further explored.

The activation of mitochondrial stress responses is associated with increased invasive and metastatic capability of breast cancer [45]. Our data indicate that mitochondrial stress responses, featured by glycolytic serine synthesis, are critical adaptations that support the proliferation of lung adenocarcinoma cell lines, particularly when the regulation of mitochondrial dynamics is hampered. Although the clinical significance of mitochondrial stress responses in lung adenocarcinoma is not well understood, the expression of PHGDH in lung adenocarcinoma, which is regulated by NRF2‐mediated signaling [36], is associated with worse patient survival [35, 36]. Moreover, EGFR‐driven trends in signaling activate glycolytic serine synthesis [46]. Therefore, glycolytic serine synthesis is an important metabolic pathway for lung adenocarcinoma and associated mitochondrial adaptations. In addition, for lung adenocarcinoma with low serine synthesis [36], the activation of mitochondrial stress responses might increase reliance on glycolytic serine synthesis and thus susceptibility to PHGDH inhibition. Collectively, PHGDH inhibitors are potential therapeutics for lung adenocarcinoma, but the optimal applications of such agents require further investigation.

Dynamin‐related protein 1 is a predicated substrate of CDK2‐cyclin A, based on proteomic analyses [47]. We confirmed the specific role of CDK2 in regulating mitotic mitochondrial fission in lung adenocarcinoma cell lines. Although CDK4/6 might compensate for CDK2 in regulating cell cycle progression [48], our data revealed that CDK4/6 is not crucial for mitosis‐related mitochondrial fragmentation in lung adenocarcinoma cell lines. The loss of CDK2 in lung adenocarcinoma cell lines cannot dramatically suppress the tumor growth [48], indicating that CDK2‐mediated DRP1 activation is dispensable for proliferation. Since other kinases, such as ERK and AKT, can regulate the activation of DRP1 in lung adenocarcinoma cell lines, the GTPase activity of DRP1 can thus be maintained even though CDK2 is depleted. Additionally, our findings suggested that combination treatments to inhibit ERK/AKT and CDK2 may potentially be effective in lung adenocarcinoma through perturbing mitochondrial dynamics, and further investigations are warranted.

Our data demonstrated that gefitinib decreased DRP1 phosphorylation in cell lines with sensitive EGFR mutation, including PC9 and HCC827, but not in cell lines with wild‐type EGFR (CL1‐0) or with resistant T790M EGFR mutation (H1975). H1975 cell is sensitive to the 3rd‐generation EGFR tyrosine kinase inhibitor, osimertinib. We confirmed that osimertinib effectively decreased DRP1 phosphorylation in H1975 cells. EGF‐driven EGFR signaling also increased DRP1 phosphorylation in CL1‐0 cells. Therefore, we herein unveiled the critical role of EGFR signaling in regulating mitochondrial fission through a multikinase framework. Several driving mechanisms other than EGFR, such as *ALK*‐related fusion oncogenes, have been uncovered in lung adenocarcinoma and can also regulate ERK and AKT signaling [49]. The mitochondrion serves as an integrated hub, sensing environmental stress and rewiring metabolic activities for appropriate cellular adaptations [50]. However, it is not well understood how these driving signaling mechanisms reshape mitochondria to support the metabolic demands of lung adenocarcinoma. Our findings thus provided the mechanistic insights bridging the driving signaling and mitochondrial reprogramming, and particularly the activation of mitochondrial fission, in lung adenocarcinoma development.

Imbalances in mitochondrial dynamics are associated human neuromuscular diseases and can result in dysfunctions in various organ systems in murine models [1]. We found that both tubular and fragmented mitochondria appear in lung adenocarcinoma cell lines, and the findings suggest that mitochondrial morphology might be flexibly altered in lung adenocarcinoma depending on the metabolic and biologic demands of the cells. Upregulated mitochondrial fusion machinery, such as OPA1, is observed in cancer cells of various origins and might be associated with worse prognosis [51]. However, it is unknown whether mitochondrial fusion is also activated for the balanced control of mitochondrial dynamics and to accelerate mitochondrial morphological alterations in lung adenocarcinoma, and further investigations are thus required for clarification.

We surmised that the upregulation of mitochondrial fission occurs early in the development of lung adenocarcinoma and investigated the clinical significance of DRP1 specifically in lung adenocarcinoma of operable stages. Whether DRP1 expression is associated with worse survival in patients with stage IV lung adenocarcinoma remains unclear and requires further examination. Screening through low‐dose computed tomography has proven effective to detect early‐stage lung cancer in the high‐risk population and to reduce lung cancer‐related mortality [52, 53]. Biomarkers associated with an increased risk of postoperative recurrence might help to identify patients who could benefit from adjuvant therapy [54]. Our findings showing the association between disease stage and DRP1 expression or activation suggest that upregulated mitochondrial fission is a critical step in promoting the progression of early‐stage lung adenocarcinoma. Therefore, although further studies are required to validate the prognostic significance of DRP1 in early‐stage lung adenocarcinoma, our results suggest that biomarkers related to mitochondrial reprogramming might help to stratify patients based on the risk of postoperative recurrence and thus warrant further exploration.

## 5 Conclusions

In conclusion, we present the functional role of DRP1 in enhancing the proliferation and invasiveness of lung adenocarcinoma cell lines and further show that multikinase regulatory molecules, including ERK, AKT, and CDK2, secure the activation of DRP1. Our findings reveal that the expression and activation of DRP1 in early‐stage lung adenocarcinoma are important features, suggesting an increased risk of postoperative recurrence. Further studies will broaden our understanding of mitochondrial functions and regulation in lung adenocarcinoma.

## Authors’ contributions

KPC and CJY conceived and designed the study. KPC, YLH, YJC, and YHJ performed the experiments. YLH and YLC provided technical support for pathologic sample processing, IHC staining, and the interpretation of IHC results. KPC, CL, and YTH performed the data analyses, and KPC, KN, MWL, SGW, JYS, YLC, and CJY provided critical discussions and comments for data interpretation. KPC wrote the manuscript, and coauthors reviewed and approved the final manuscript.

## Conflict of interest

The authors declare no conflict of interest.

## Supporting information


**Fig S1.** (in relation to Fig. 1). Analysis of data from TCGA‐LUAD to evaluate the prognostic significance of *DNM1L* expression in lung adenocarcinoma.
**Fig S2.** (in relation to Fig. 1). DRP1 expression and activation are associated post‐operative recurrence in early stage lung adenocarcinoma.
**Fig S3.** (in relation to Fig. 1). DRP1 expression and activation are significantly associated with post‐operative recurrence of lung adenocarcinoma.
**Fig S4.** (in relation to Fig. 2). DRP1 depletion increases mitophagy at baseline and after mitochondrial damage in lung adenocarcinoma cell lines.
**Fig S5.** (in relation to Fig. 3). DRP1 expression and activation are associated with proliferation and disease extent of lung adenocarcinoma.
**Fig S6.** (in relation to Fig. 3). The effects of oxidative phosphorylation inhibition to proliferation and invasion of lung adenocarcinoma.
**Fig S7.** (in relation to Fig. 4). Gefitinib decreases DRP1 phosphorylation in sensitive lung adenocarcinoma cell lines.
**Fig S8.** (in relation to Fig. 5). Transduction of the lentiCRISPRv2 vector did not alter the cell cycle progression.
**Fig S9.** (in relation to Fig. 5). The effects of various CDK inhibitors to cell cycle progression.
**Fig S10.** (in relation to Fig. 6). CDK2 regulates DRP1 phosphorylation during cell cycle.
**Fig S11.** (in relation to Fig. 6). CDK2 regulates DRP1 phosphorylation during cell cycle.

**Table S1.** Information of the reagents and antibodies.

**Table S2.** Nucleotide sequences for qPCR primer pairs and guide RNA for CRISPR/Cas9 knockout.

**Table S3.** Clinical characteristics of the study population.

**Table S4.** Multi‐variate Cox proportional hazard ratio model for 5‐year post‐operative recurrence.
