# Supplementary material for: Multi‐kinase framework promotes proliferation and invasion of lung adenocarcinoma through activation of dynamin‐related protein 1
Source: Mol Oncol. 2020 Dec 11;15(2):560–78. doi: 10.1002/1878-0261.12843 (PMC7858280; doi:10.1002/1878-0261.12843)

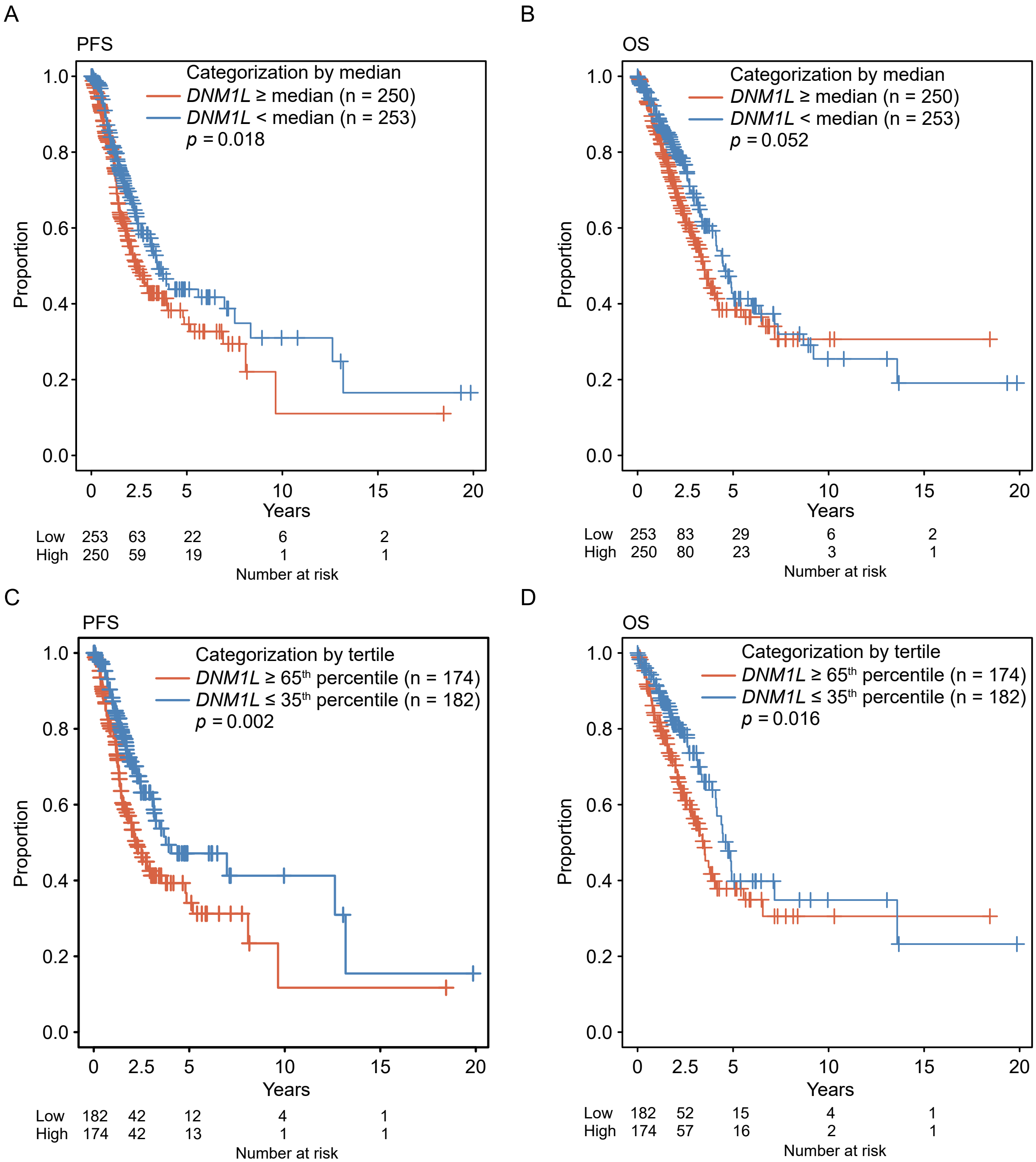

Fig. S1 (in relation to Fig. 1). Analysis of data from TCGA-LUAD to evaluate the prognostic significance of *DNM1L* expression in lung adenocarcinoma. The results showed that *DNM1L* expression above the median is associated with significantly decreased progression-free survival (PFS) (A), and a non-significant trend of decreased overall survival (OS) (B). Further evaluation revealed that tumors with *DNM1L* expression higher than the 65 percentile, compared with tumors with *DNM1L* expression less than the 35 percentile, were significantly associated with decreased PFS (C) and OS (D). The survival differences were all compared using a log-rank test.

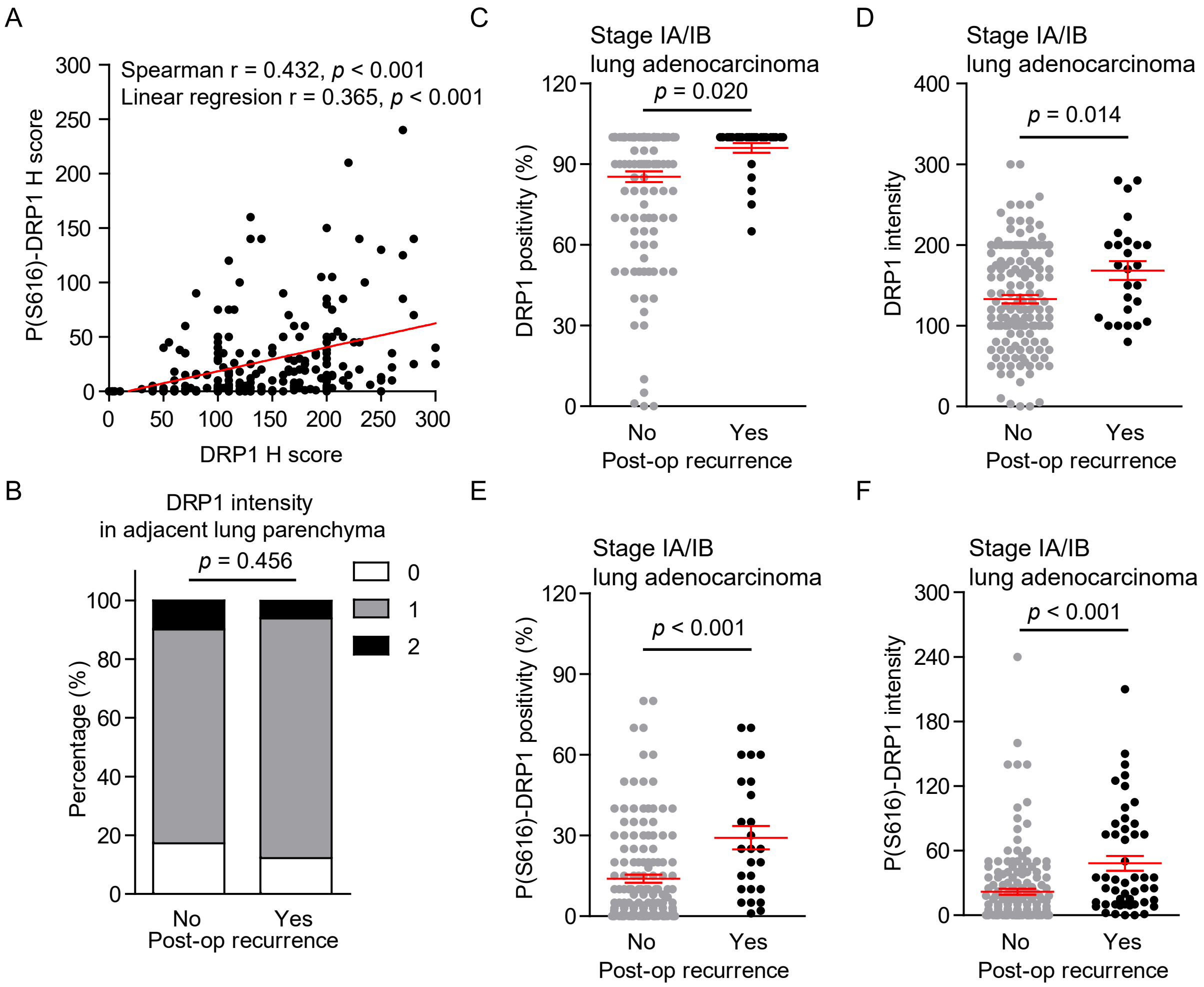

Fig. S2 (in relation to Fig. 1). DRP1 expression and activation are associated post-operative recurrence in early stage lung adenocarcinoma. (A) The correlation between DRP1 H-score and phosphorylated DRP1 H-score, determined based on immunohistochemistry staining using surgical samples. The correlation coefficient (r) was calculated by Spearman correlation or linear regression analysis. (B) The DRP1 expression in adjacent non-cancerous tissues is not significantly associated with post-operative recurrence. The difference was compared using a Pearson's chi-square test. The association between post-operative recurrence and DRP1 expression (C and D) or activation (E and F), quantified by positivity and H-score, in stage I lung adenocarcinoma. The data in C, D, E and F is presented as mean±standard error, and the differences were compared using a Mann Whitney U test.

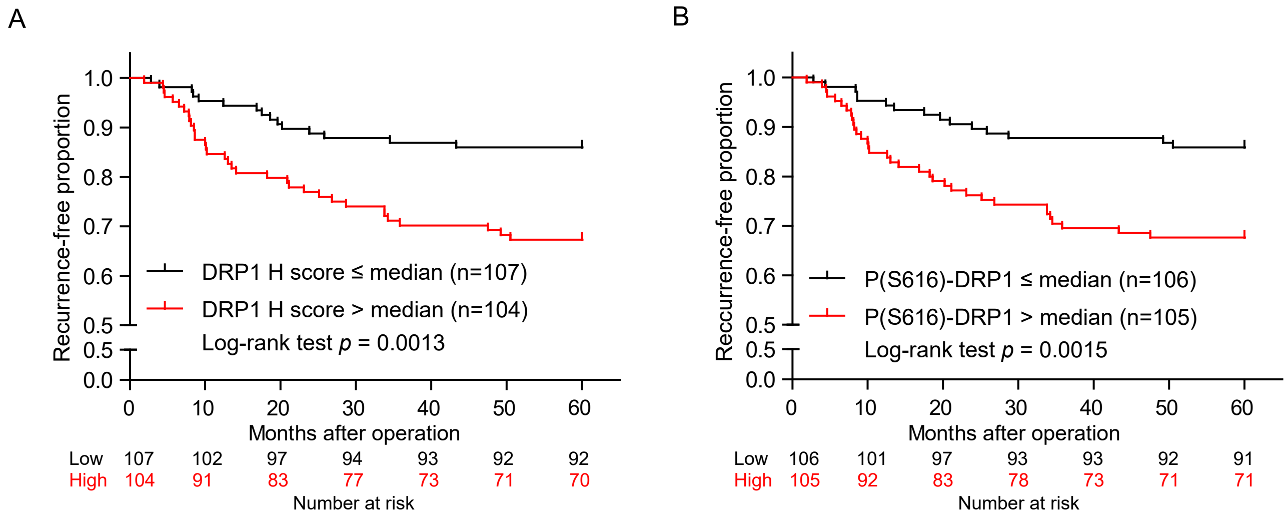

Fig. S3 (in relation to Fig. 1). DRP1 expression and activation are significantly associated with post-operative recurrence of lung adenocarcinoma. The study population was dichotomized based on the median of DRP1 or P(S616)-DRP1 H-scores, and Kaplan-Meier curves were plotted for evaluating the recurrence-free survival. The results demonstrated that both (A) DRP1 and (B) P(S616)-DRP1 expression in lung adenocarcinoma were significantly associated with decreased recurrence-free survival.

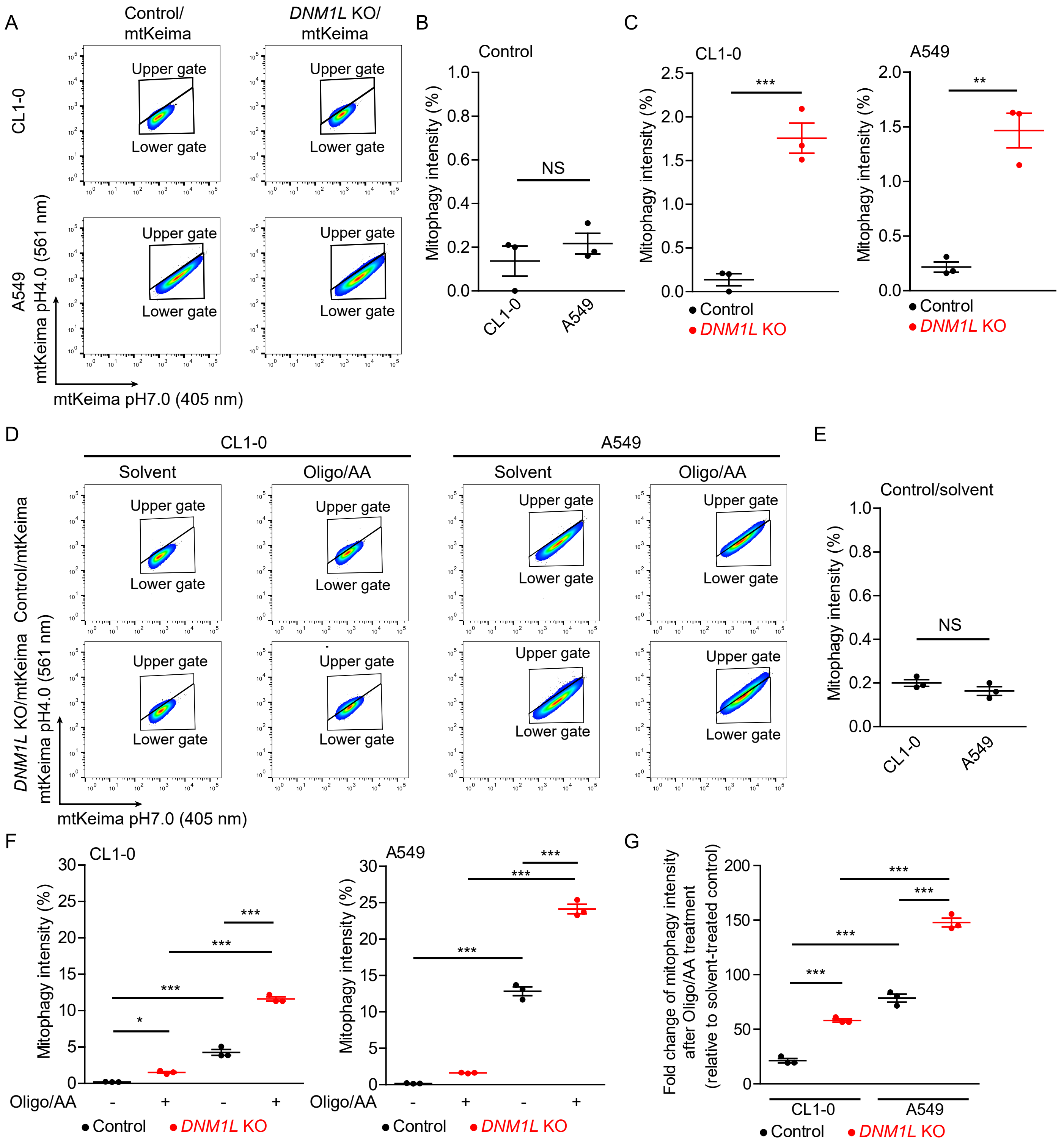

Fig. S4 (in relation to Fig. 2). DRP1 depletion increases mitophagy at baseline and after mitochondrial damage in lung adenocarcinoma cell lines. (A) Representative flow cytometric analysis demonstrating the measurement of mitophagy intensity (the percentage of cells in the upper gate) in lung adenocarcinoma cell lines at baseline. (n = 3 technical repeats). The mitophagy intensity was not significantly different between CL1-0 and A549 (B), and depletion of DRP1 led to modestly but significantly increased mitophagy intensity (C). (D) Representative flow cytometric analysis showing mitophagy induction using oligomycin and antimycin A (Olgio/AA) (treatment for 24 hours; n = 3 technical repeats). The mitophagy intensity between CL1-0 and A549 was similar after solvent treatment (E), and *DNM1L* KO CL1-0 and A549 had significanlty higher mitophagy intensity than the control (F). The fold change of Oligo/AA-induced mitophagy is signficantly higher in A549 cells, either control or *DNM1L*-KO, than in CL1-0 cells (G). Data in B, C, E, F and G is presented as mean±standard error. The differences were compared using a Student's t test (B, C, and E), or one-way ANOVA (F and G; NS non-significant, * $p < 0.05$, *** $p < 0.001$)

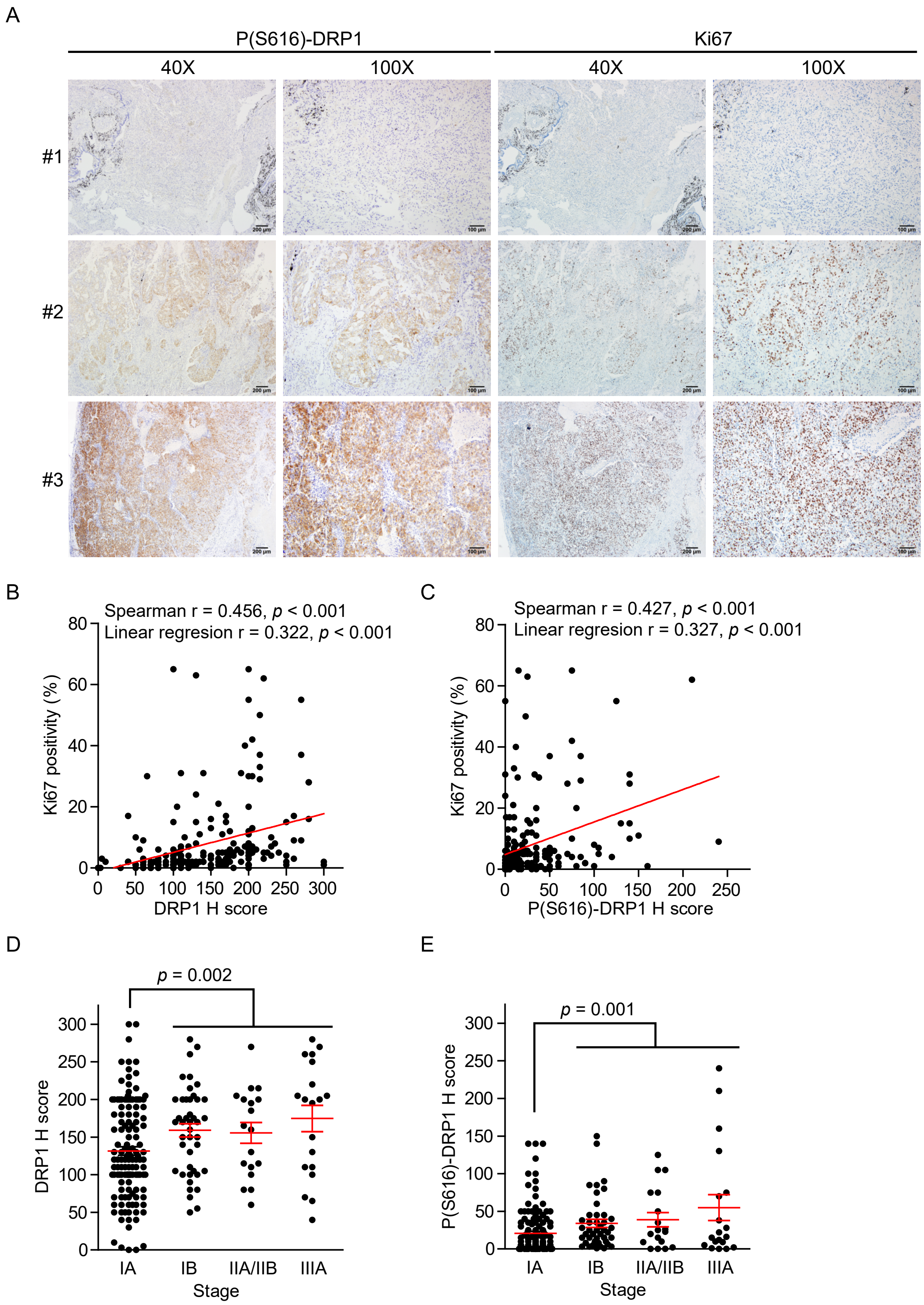

Fig. S5 (in relation to Fig. 3). DRP1 expression and activation are associated with proliferation and disease extent of lung adenocarcinoma. (A) Representative immunohistochemistry staining of phosphorylated DRP1 and Ki67 using sections from paraffin-embedded lung adenocarcinoma samples. (B and C) The correlation between Ki67 positivity and the H-score of DRP1 or phosphorylated DRP1. The correlation coefficient (r) was calculated by Spearman correlation or linear regression analysis. (D and E) The association of H-score of DRP1 or phosphorylated DRP1 and disease stage of lung adenocarcinoma. Data in D and E is presented as mean±standard error, and the differences were compared using a Student's t test.

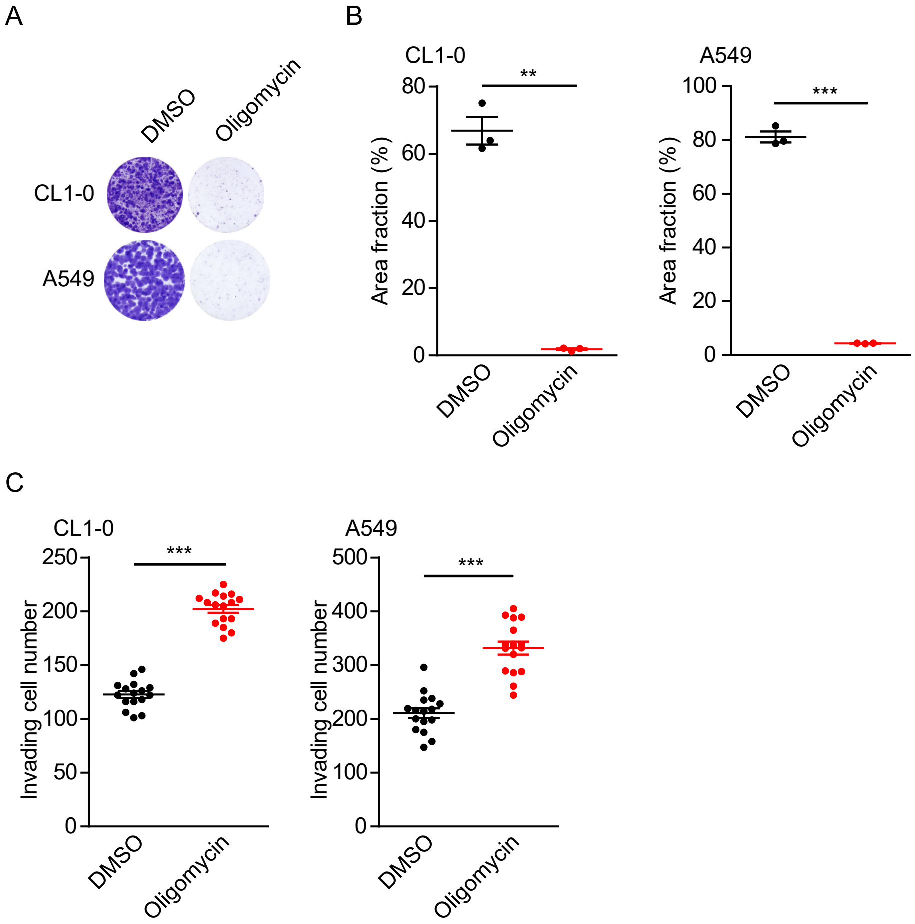

Fig. S6 (in relation to Fig. 3). The effects of oxidative phosphorylation inhibition to proliferation and invasion of lung adenocarcinoma. (A) The representative well images of colony formation assay. (B) The quantification of the colony formation assay. Each dot indicates a well in a 6-well culture plate (n = 2 technical repeats). (C) The quantification of invasion assay through counting the invading cell number. Each dot indicates a fluorescent microscopy field under 10X objective (n = 2 technical repeats). Data in B and C is presented as mean±standard error, and the differences were compared using a Student's t test (** $p < 0.01$, *** $p < 0.001$).

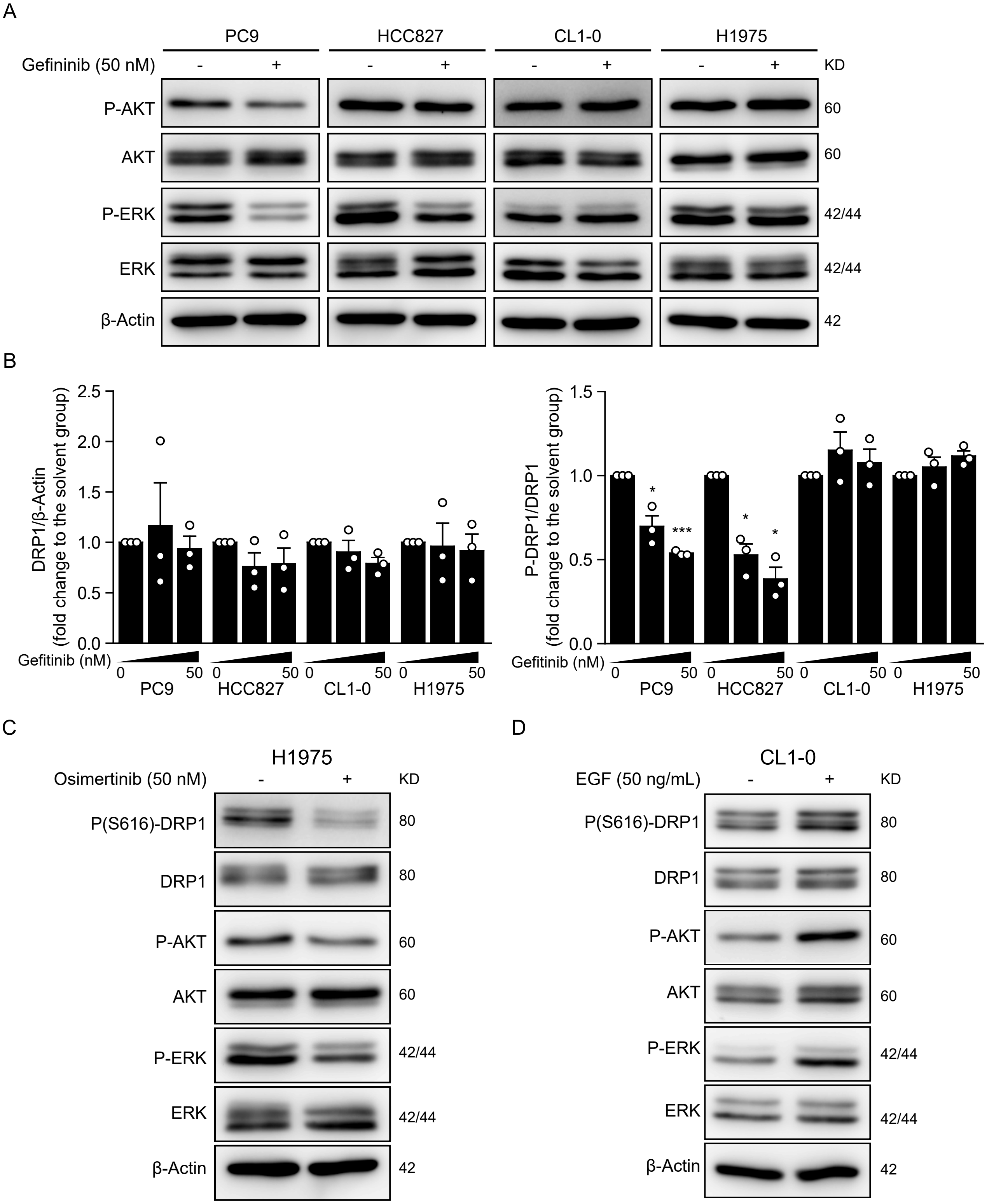

Fig. S7 (in relation to Fig. 4). Gefitinib decreases DRP1 phosphorylation in sensitive lung adenocarcinoma cell lines. (A) Representative immunoblots showing the effects of gefinitib in inhibiting phosphorylation of ERK and AKT after treatment for 24 hours (n = 2 technical repeats). (B) Densitometry quantification of DRP1 and phosphorylated DRP1 expression after DMSO or gefitinib treatment (10 nM or 50 nM) using the immunoblots. Each dot indicates one immunoblot replicate. Data is presented as mean±standard error, and the differences were compared using a Student's t test (* $p < 0.05$, *** $p < 0.001$). (C) Immnoblots showing the effect of osimertinib on DRP1 phosphorylation in H1975 (24-hour treatment; n = 2 technical repeats). (D) Immunoblots showing the phosphorylation of DRP1 in CL1-0 after epidermal growth factor (EGF) stimulation for 30 minutes (n = 2 technical repeats).

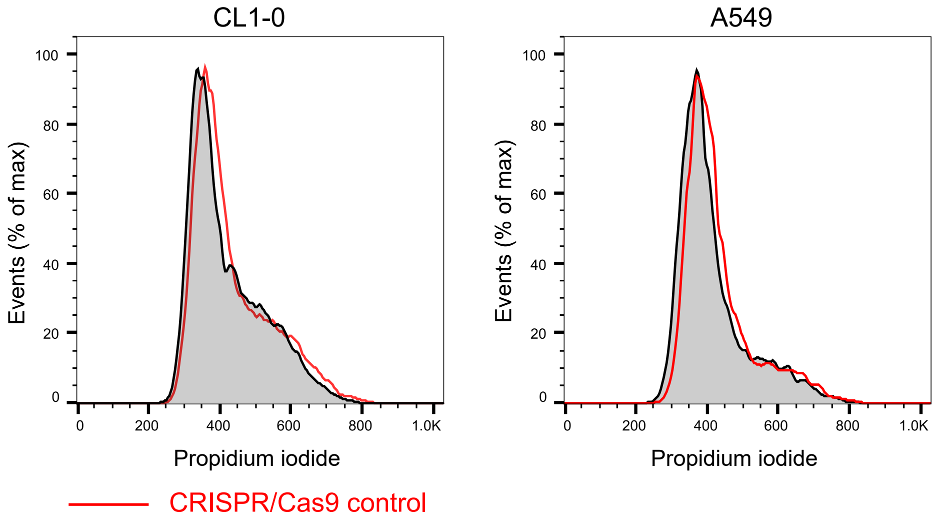

Fig. S8 (in relation to Fig. 5). Transduction of the lentiCRISPRv2 vector did not alter the cell cycle progression. Representative flow cytometric histogram showing cell cycle progression of the cells after the lentiCRISPRv2 vector transduction(red line, the CRISPR/Cas9 control cells) and that of the cells without transduction (black line and grey-color shaded area) (n = 3 technical repeats).

A

CDK inhibitors	Target				References
	CDK4/6	CDK2	CDK5	CDK1	
Roscovitine		+	+	+	Meijier L, et al. *Eur J Biochem* 1997; 243: 527
PHA-793887		+	+	+	Brasca MG, et al. *Bioorg Med Chem* 2010; 18: 1844
Ro-3306				+	Vassilev LT, et al. *Proc Natl Acad Sci USA* 2006; 103: 10660
Palbociclib	+				Fry DW, et al. *Mol Cancer Ther* 2004; 3: 1427

B

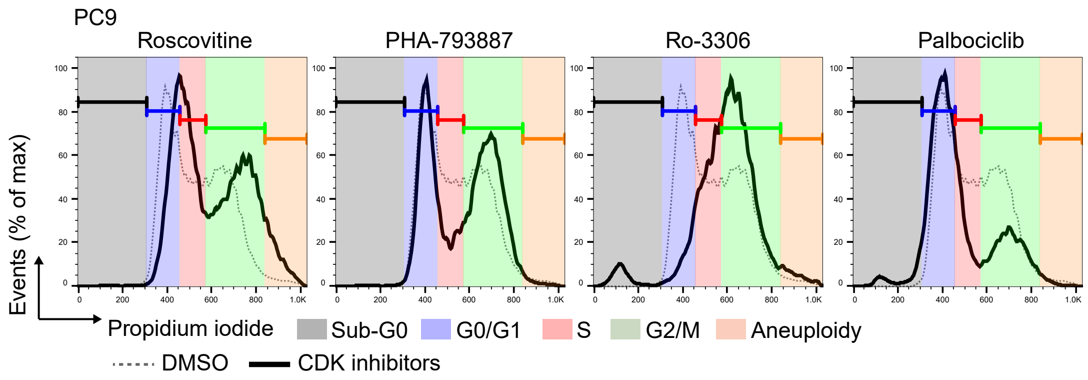

C

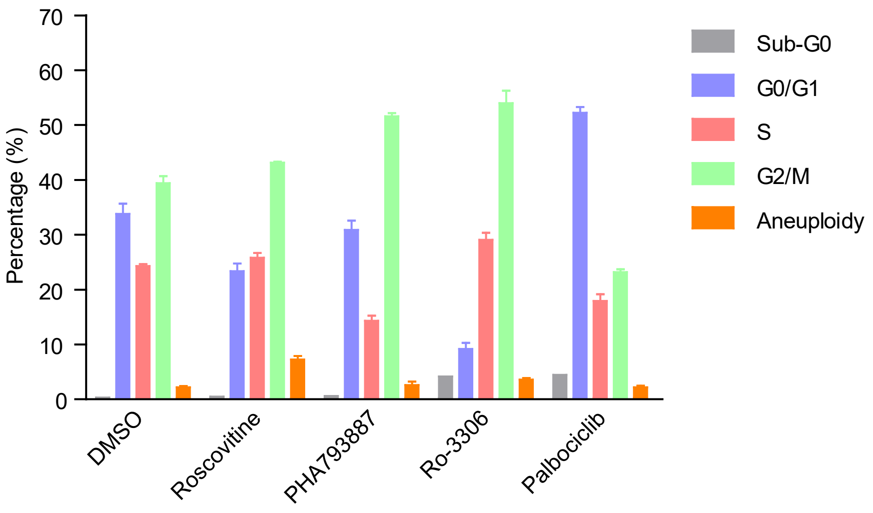

	p value (compared with DMSO group)				
	Sub-G0	G0/G1	S	G2/M	Aneuploidy
Roscovitine		*			**
PHA793887	*		***	**	
Ro-3306	***	***	*	**	*
Palbociclib	**	***	**	***	

Fig. S9 (in relation to Fig. 5). The effects of various CDK inhibitors to cell cycle progression. (A) A table summarizes the CDKs targeted by different CDK inhibitors based on previous literatures. (B) Representative flow cytometric histogram showing cell cycle progression under different CDK inhibitors in the PC9 cell line (24-hour treatment; n = 2 technical repeats). (C) Percentages of cells at different phases of cell cycle under CDK inhibitors in the PC9 cell line. Data is presented as mean±standard error, and the differences were compared using a Student's t test (* $p < 0.05$, ** $p < 0.01$, *** $p < 0.001$).

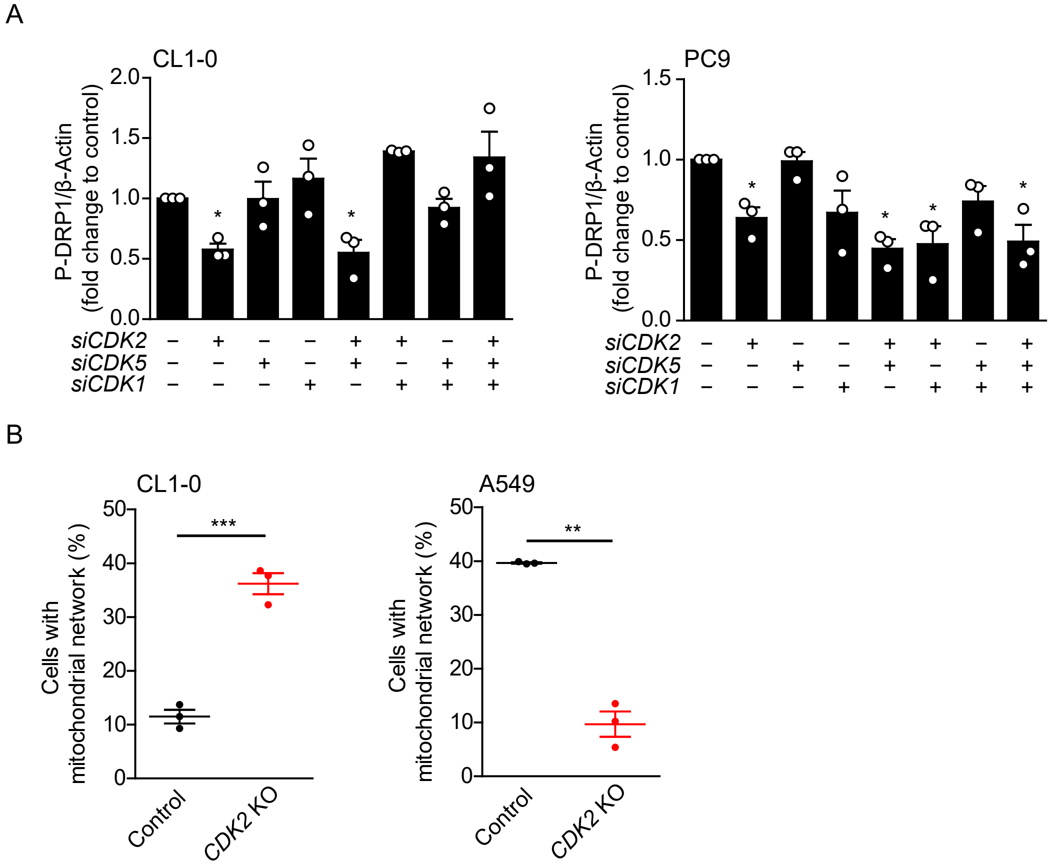

Fig. S10 (in relation to Fig. 6). CDK2 regulates DRP1 phosphorylation during cell cycle. (A) Desitometric quantification of phosphorylated DRP1 expression after *CDK2*, *CDK5* and *CDK1* knock-down, using immunoblots. Each dot indicates one immunoblot replicate. (B) Quantification of mitochondrial morphology using confocal imaging. Each dot indicates a glass-bottom dish, and 3 high power fields, constituted by 3 x 3 tile images per dish were used for quantification. Each high power field contains more than 100 cells. Data is presented as mean±standard error, and the differences were compared using a Student's t test (* $p < 0.05$, ** $p < 0.01$, *** $p < 0.001$).

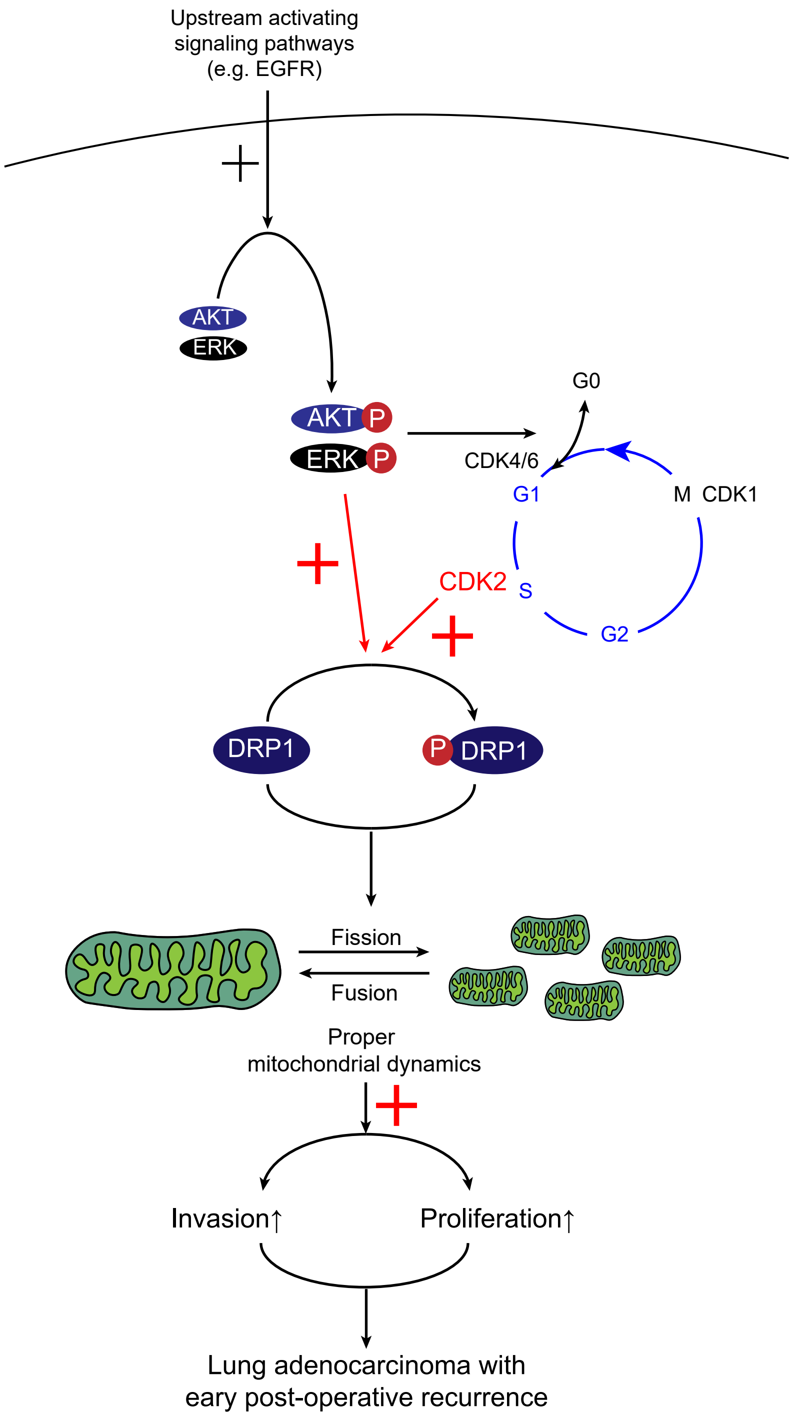

Fig. S11 (in relation to Fig. 6). Multi-kinase regulation of DRP1-mediated mitochondrial fission promotes proliferation and invasion of lung adenocarcinoma.

Uncropped membranes of immunoblots

Fig. 2A

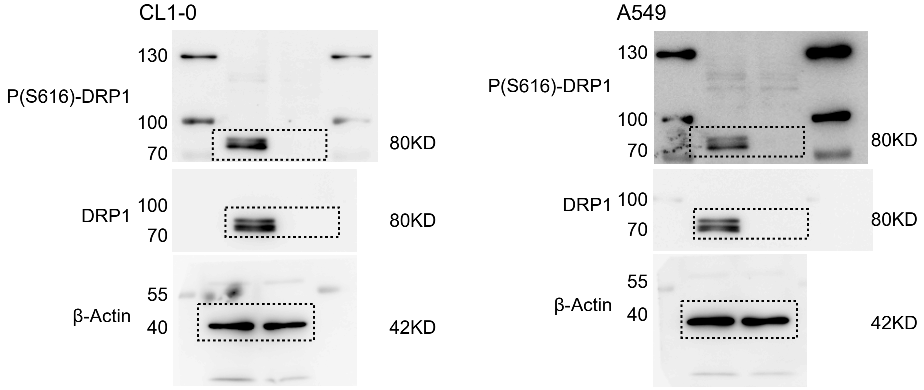

Fig. 2G

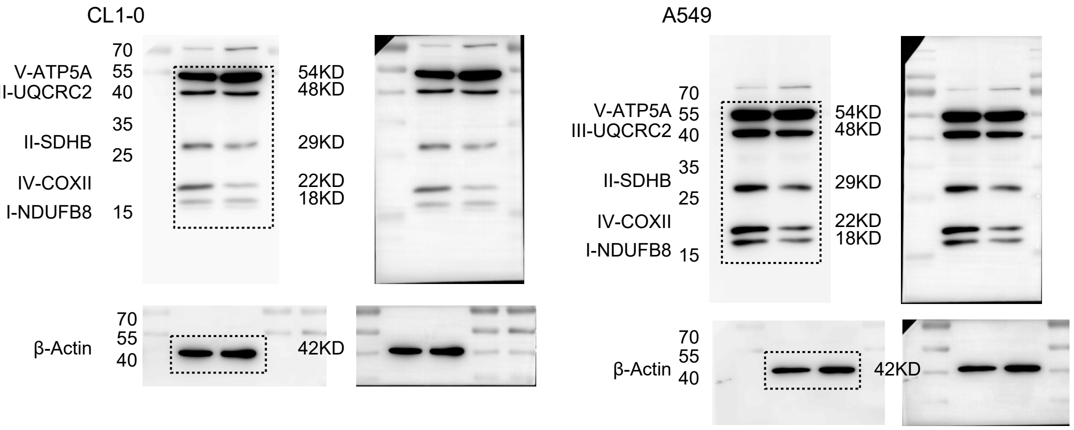

Fig. 3G

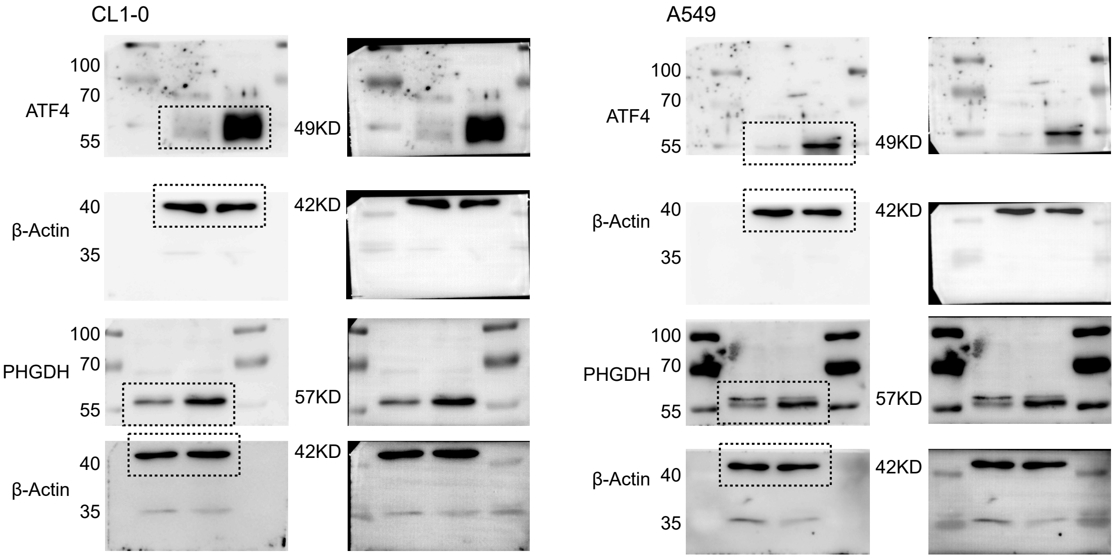

Fig. 4A

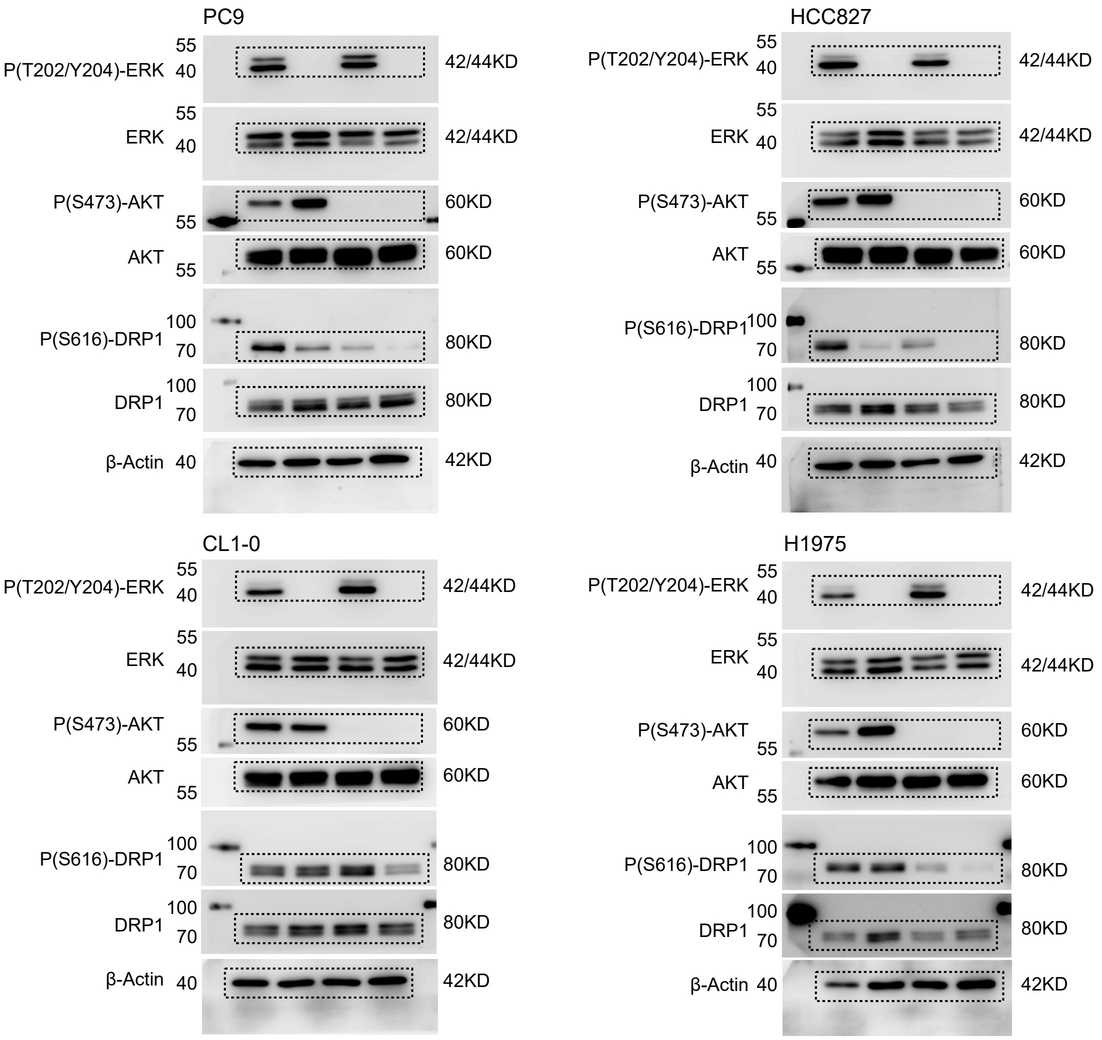
PC9
HCC827
P(T202/Y204)-ERK
ERK
P(S473)-AKT
AKT
P(S616)-DRP1
DRP1
β-Actin
55
40
100
70
42/44KD
60KD
80KD
42KD
CL1-0
H1975

Fig. 4B

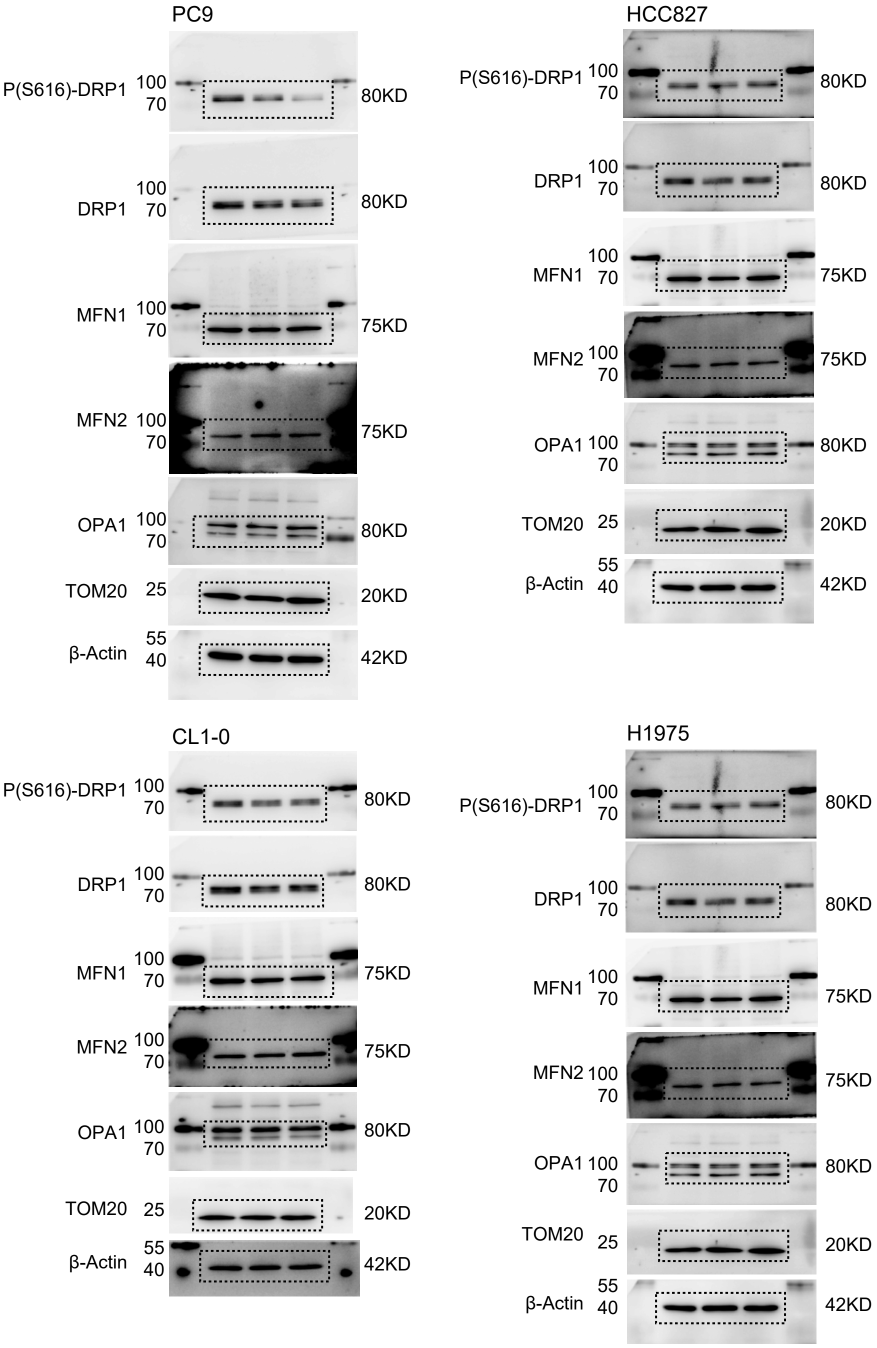
PC9
P(S616)-DRP1 100 70 80KD
DRP1 100 70 80KD
MFN1 100 70 75KD
MFN2 100 70 75KD
OPA1 100 70 80KD
TOM20 25 20KD
β-Actin 55 40 42KD
HCC827
P(S616)-DRP1 100 70 80KD
DRP1 100 70 80KD
MFN1 100 70 75KD
MFN2 100 70 75KD
OPA1 100 70 80KD
TOM20 25 20KD
β-Actin 55 40 42KD
CL1-0
P(S616)-DRP1 100 70 80KD
DRP1 100 70 80KD
MFN1 100 70 75KD
MFN2 100 70 75KD
OPA1 100 70 80KD
TOM20 25 20KD
β-Actin 55 40 42KD
H1975
P(S616)-DRP1 100 70 80KD
DRP1 100 70 80KD
MFN1 100 70 75KD
MFN2 100 70 75KD
OPA1 100 70 80KD
TOM20 25 20KD
β-Actin 55 40 42KD

Fig. 5D

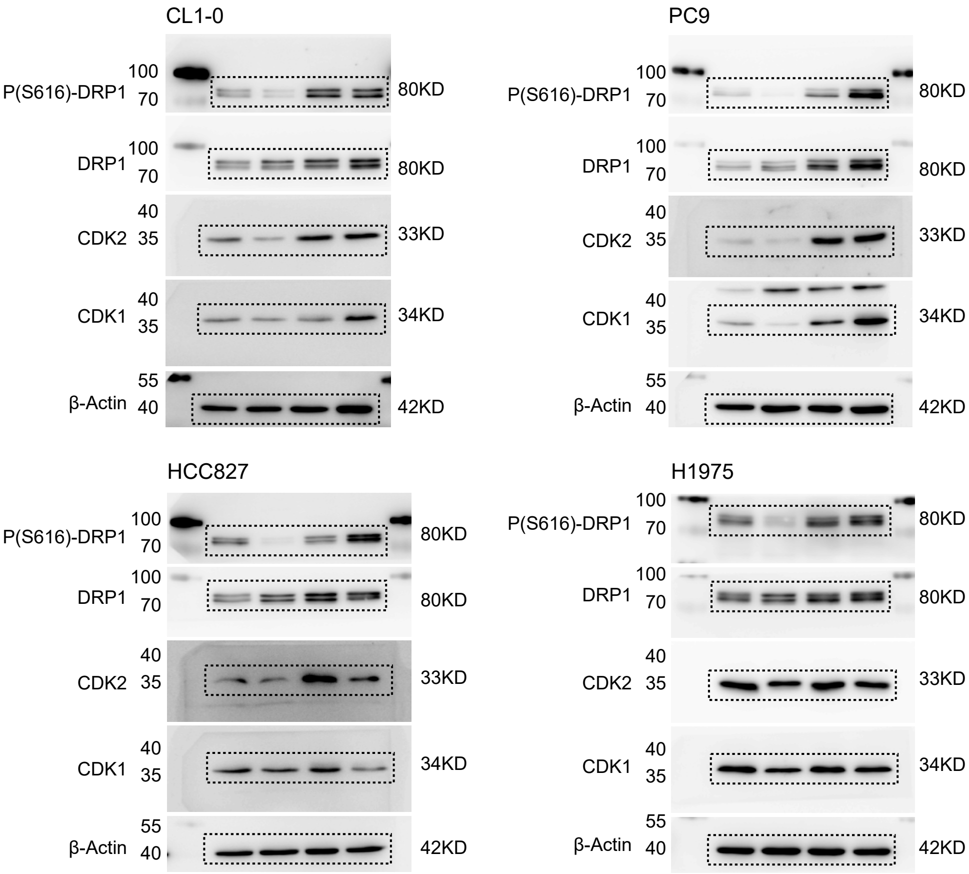
CL1-0
PC9
HCC827
H1975
P(S616)-DRP1
DRP1
CDK2
CDK1
β-Actin
100
70
40
35
55
80KD
33KD
34KD
42KD

Fig. 5F

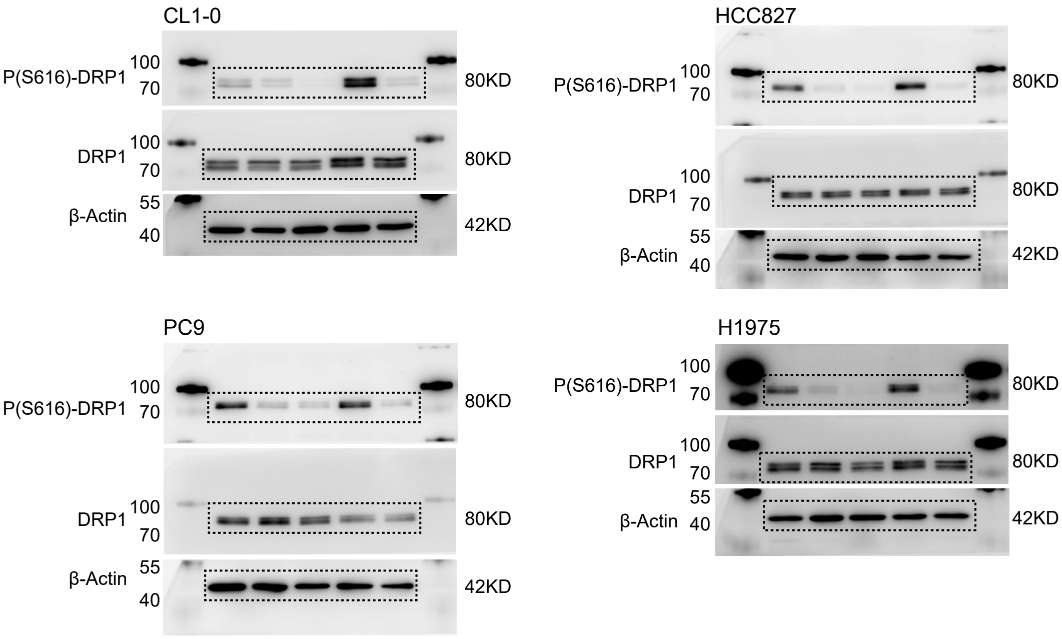
CL1-0
HCC827
PC9
H1975
P(S616)-DRP1
DRP1
β-Actin
100
70
55
40
80KD
42KD

Fig. 6A

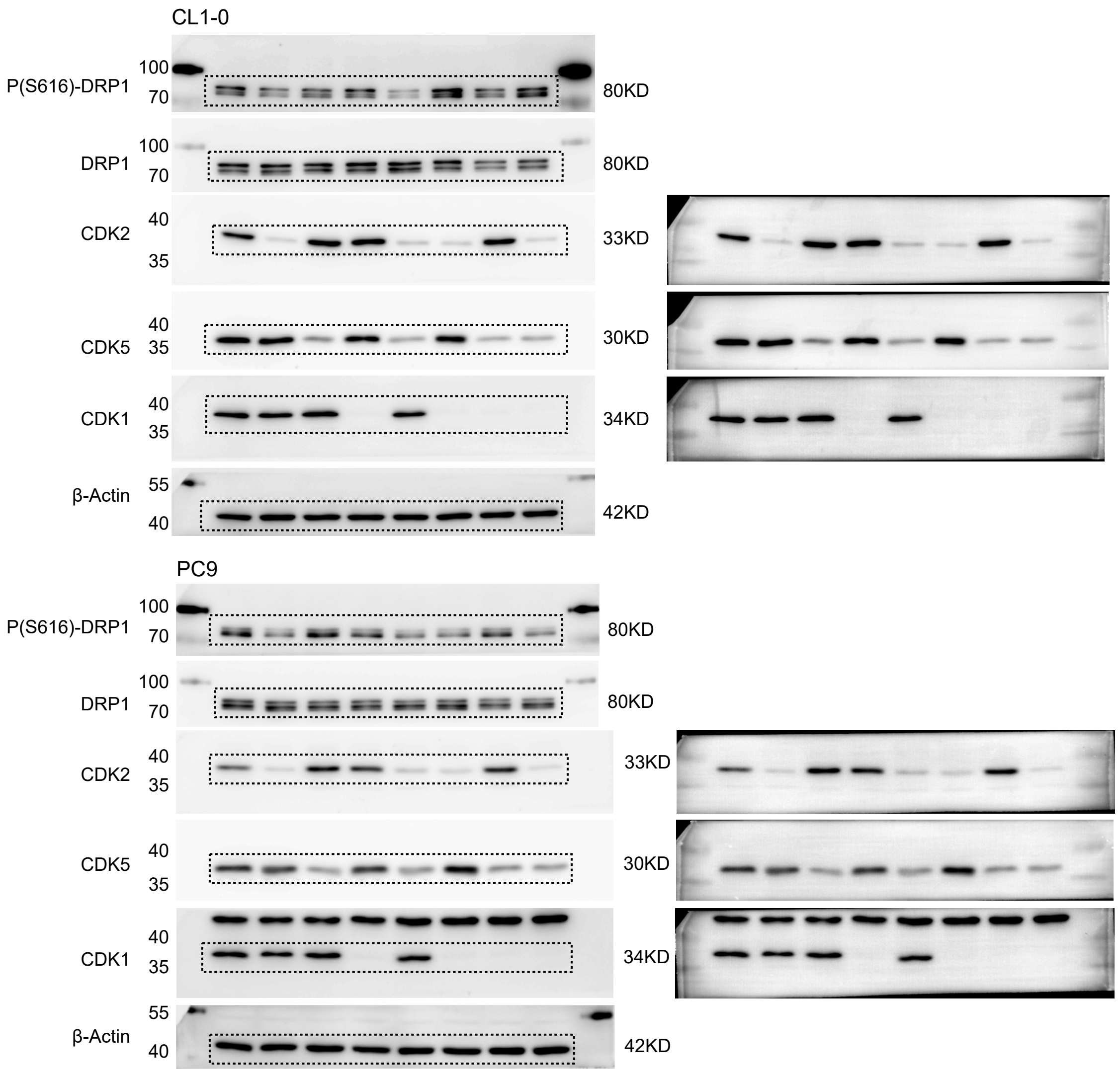
CL1-0
P(S616)-DRP1
100
70
80KD
DRP1
100
70
80KD
CDK2
40
35
33KD
CDK5
40
35
30KD
CDK1
40
35
34KD
β-Actin
55
40
42KD
PC9
P(S616)-DRP1
100
70
80KD
DRP1
100
70
80KD
CDK2
40
35
33KD
CDK5
40
35
30KD
CDK1
40
35
34KD
β-Actin
55
40
42KD

Fig. 6B

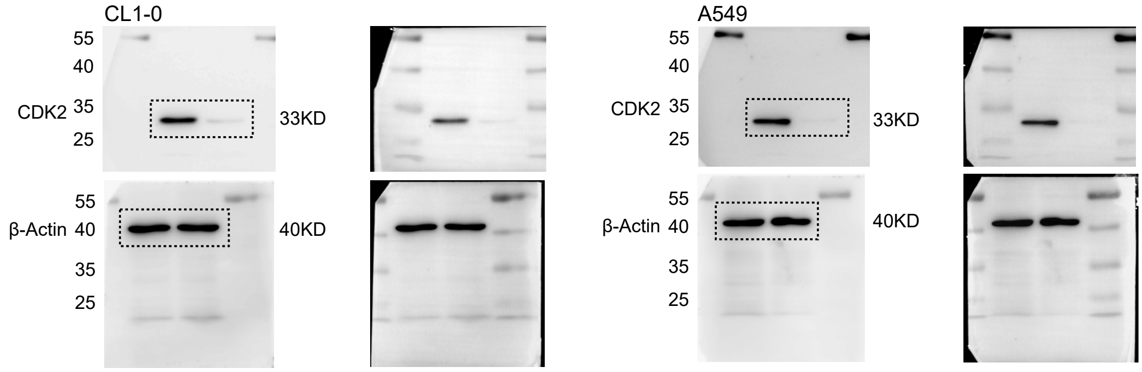
CL1-0
CDK2
55
40
35
25
33KD
β-Actin
55
40
35
25
40KD
A549
CDK2
55
40
35
25
33KD
β-Actin
55
40
35
25
40KD

Fig. 6C

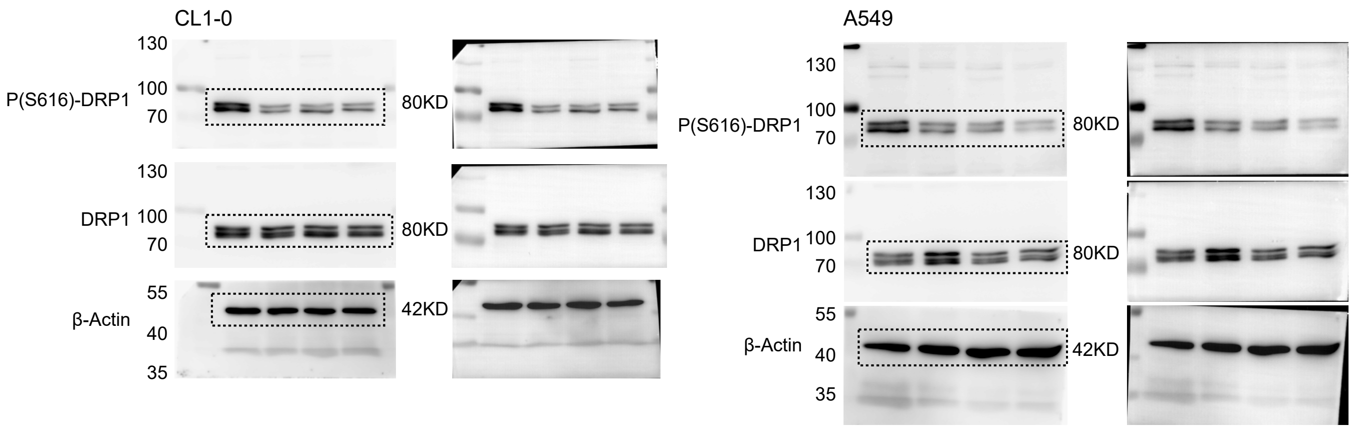
CL1-0
130
P(S616)-DRP1
100
70
80KD
130
DRP1
100
70
80KD
55
β-Actin
40
35
42KD
A549
130
P(S616)-DRP1
100
70
80KD
130
DRP1
100
70
80KD
55
β-Actin
40
35
42KD

Fig. 6F

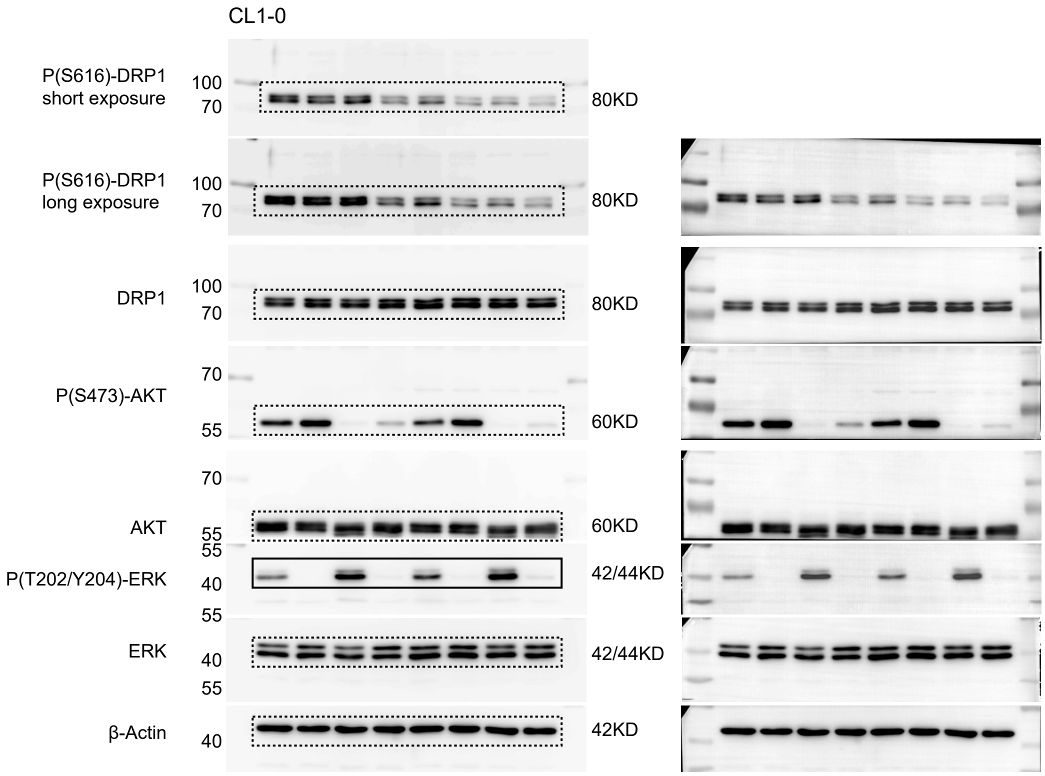
CL1-0
P(S616)-DRP1
short exposure
100
70
80KD
P(S616)-DRP1
long exposure
100
70
80KD
DRP1
100
70
80KD
P(S473)-AKT
70
55
60KD
AKT
70
55
60KD
P(T202/Y204)-ERK
55
40
42/44KD
ERK
55
40
42/44KD
β-Actin
55
40
42KD

Fig. 6F (continued)

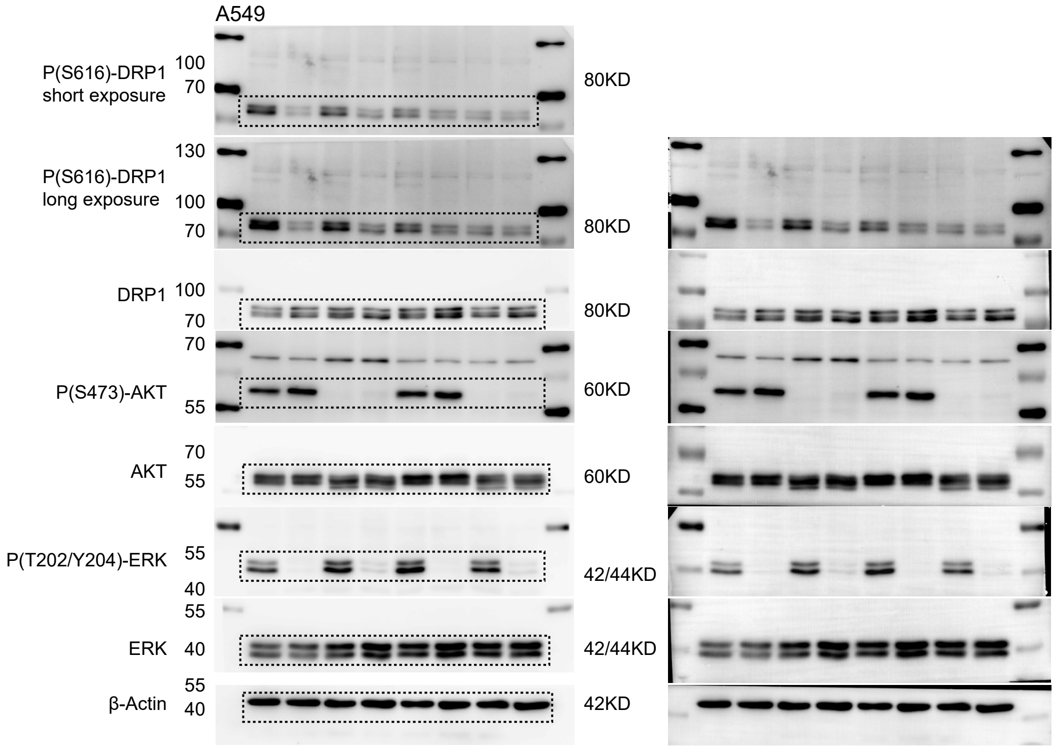

Fig. S7A

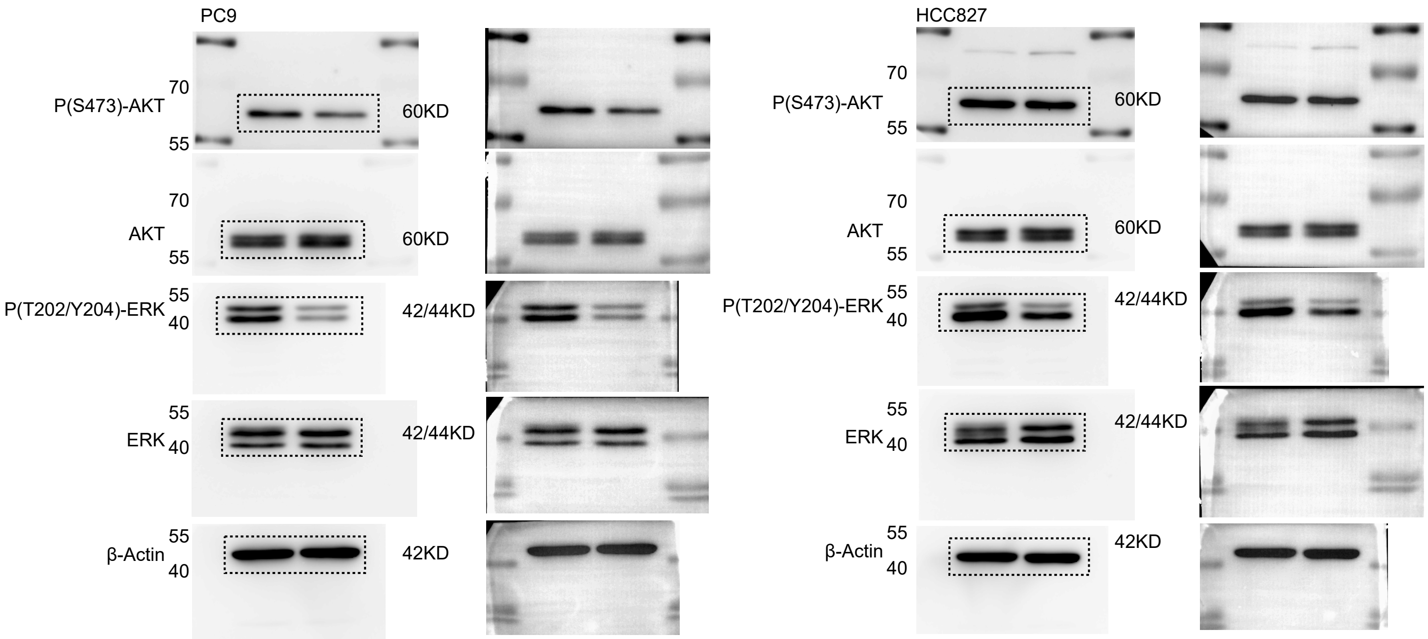

Fig. S7A (continued)

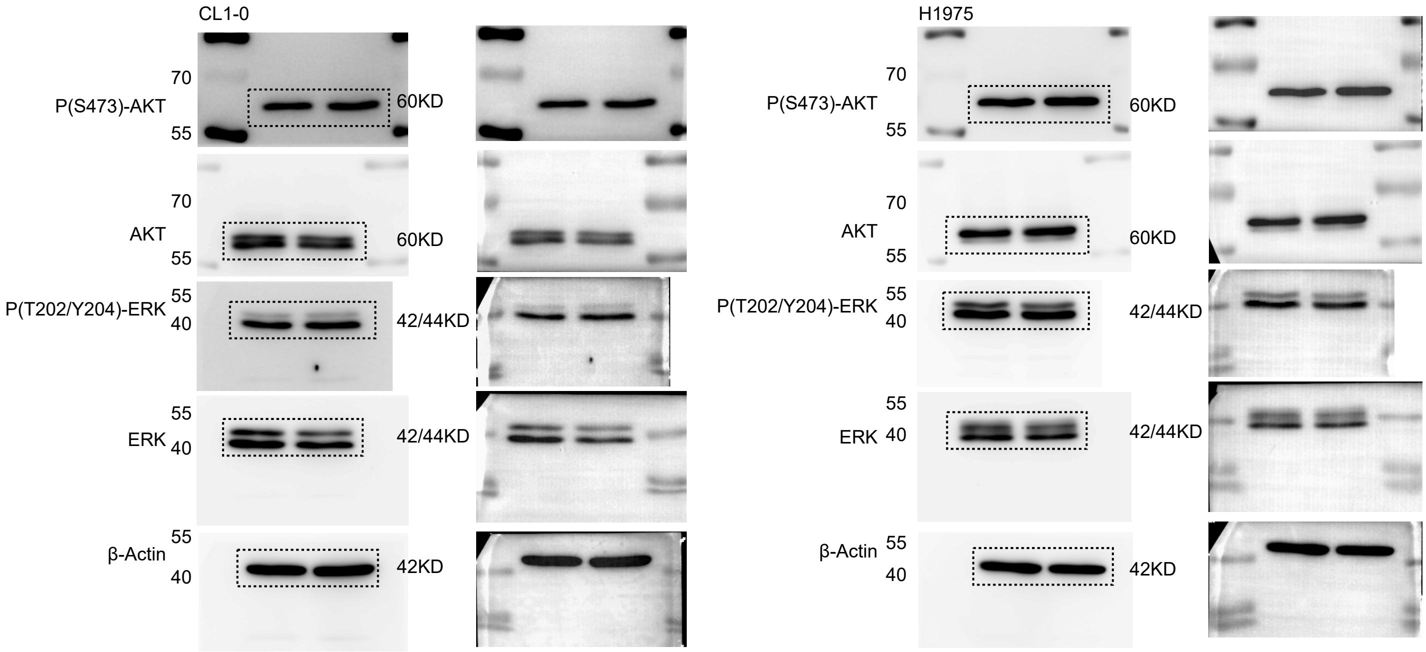

Fig. S7C

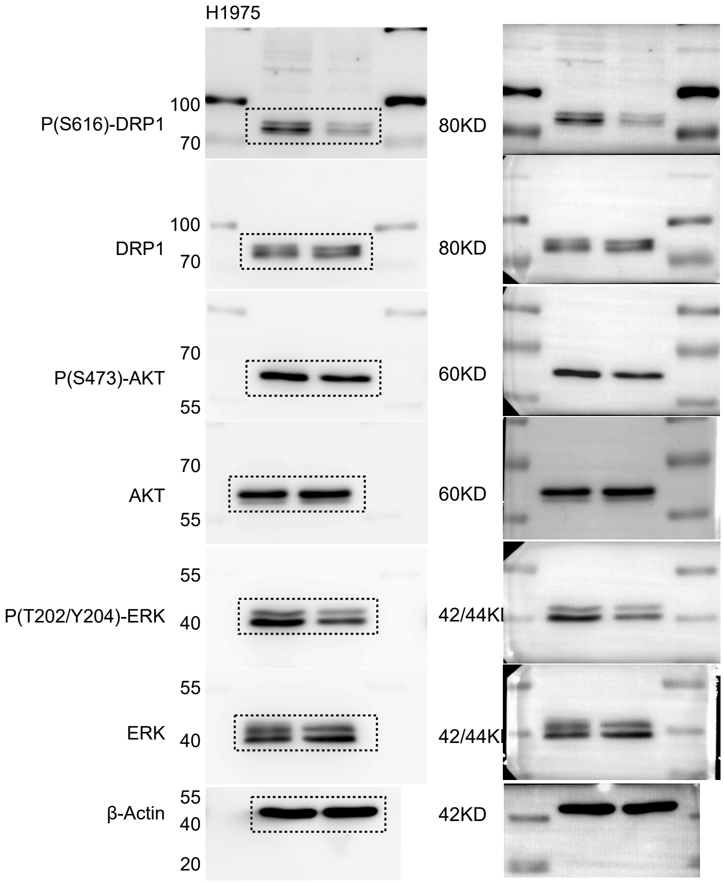

Fig. S7D

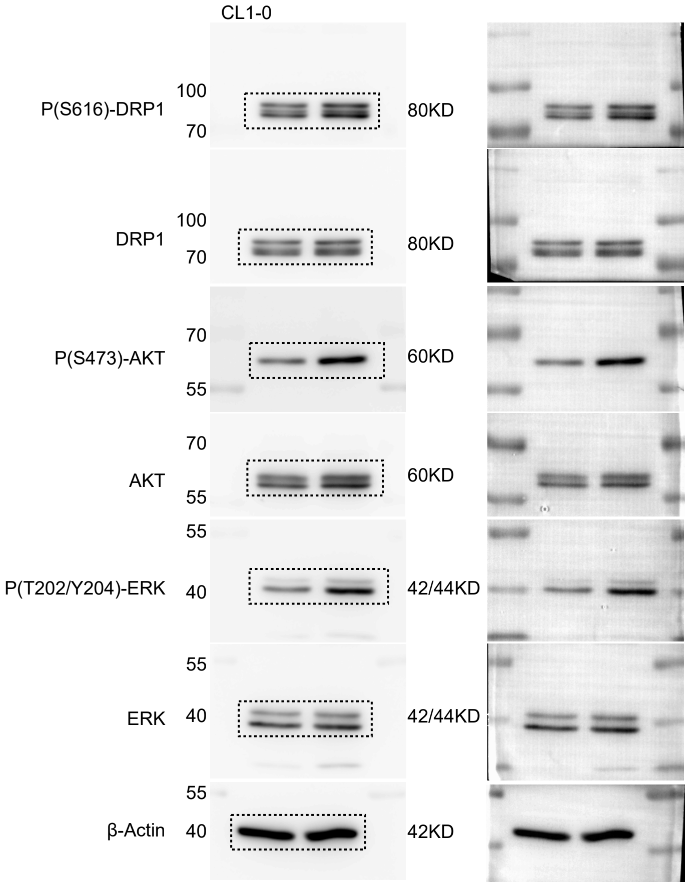

Supplement: Supplementary file 1 — Fig S1. (in relation to Fig. 1). Analysis of data from TCGA‐LUAD to evaluate the prognostic significance of DNM1L expression in lung adenocarcinoma. Fig S2. (in relation to Fig. 1). DRP1 expression and activation are associated post‐operative recurrence in early stage lung adenocarcinoma. Fig S3. (in relation to Fig. 1). DRP1 expression and activation are significantly associated with post‐operative recurrence of lung adenocarcinoma. Fig S4. (in relation to Fig. 2). DRP1 depletion increases mitophagy at baseline and after mitochondrial damage in lung adenocarcinoma cell lines. Fig S5. (in relation to Fig. 3). DRP1 expression and activation are associated with proliferation and disease extent of lung adenocarcinoma. Fig S6. (in relation to Fig. 3). The effects of oxidative phosphorylation inhibition to proliferation and invasion of lung adenocarcinoma. Fig S7. (in relation to Fig. 4). Gefitinib decreases DRP1 phosphorylation in sensitive lung adenocarcinoma cell lines. Fig S8. (in relation to Fig. 5). Transduction of the lentiCRISPRv2 vector did not alter the cell cycle progression. Fig S9. (in relation to Fig. 5). The effects of various CDK inhibitors to cell cycle progression. Fig S10. (in relation to Fig. 6). CDK2 regulates DRP1 phosphorylation during cell cycle. Fig S11. (in relation to Fig. 6). CDK2 regulates DRP1 phosphorylation during cell cycle. [file MOL2-15-560-s001.pdf]
