# Supplementary material for: Multi‐kinase framework promotes proliferation and invasion of lung adenocarcinoma through activation of dynamin‐related protein 1
Source: Mol Oncol. 2020 Dec 11;15(2):560–78. doi: 10.1002/1878-0261.12843 (PMC7858280; doi:10.1002/1878-0261.12843)
Supplement: Supplementary file 2 — Table S1. Information of the reagents and antibodies. [file MOL2-15-560-s002.docx]

**Table S1**. Information of the reagents and antibodies

| ***Chemicals for cell experiments*** | | | |
| --- | --- | --- | --- |
| Reagents | Catalog # | | Company |
| Thymidine | T1895 | | Sigma,  St. Louis, MO |
| Nocodazole | M1404 | |  |
| NCT503 | SML1659 | |  |
| Oligomycin | 75351 | |  |
| Antimycin A | A8674 | |  |
| Epidermal growth factor | AF-100-15 | | PeproTech, |
| Roscovitine | 9885 | | Cell Signaling,  Beverly, MA |
| Gefitinib | S0125 | | Selleckchem,  Houston, TX |
| Osimertinib | S7297 | |  |
| Palbociclib | S1579 | |  |
| PHA-793887 | S1487 | |  |
| Ro-3306 | S7747 | |  |
| PD184352 | S1020 | |  |
| MK2206 | S1078 | |  |
| ***Reagents for small interfering RNA (siRNA) transfection*** | | | |
| Reagents | Catalog # | | Company |
| Lipofectamine® RNAiMAX transfection reagent | 13778150 | | ThermoFisher, Waltham, MA |
| Non-targeting siRNA | D-001206-14-05 | | Dharmacon,  Lafayette, CO |
| *CDK1* siRNA | M-003224-03-0005 | |  |
| *CDK2* siRNA | M-003236-04-0005 | |  |
| *CDK5* siRNA | M-003239-01-0005 | |  |
| ***Staining for confocal microscopy*** | | | |
| Reagents | Catalog # | | Company |
| MitoTracker Green FM | M7514 | | Invitrogen,  Waltham, MA |
| Hoechst 33342 | H3570 | |  |
| ***Immunohistochemistry staining*** | | | |
| Antibody | Dilution | Catalog # | Company |
| DRP1 | 1:100 | ab56788 | Abcam,  Cambridge, MA |
| P(S616)-DRP1 | 1:40 | 3455 | Cell Signaling,  Beverly, MA |

**Table S1**. (continued)

| ***Immunohistochemistry staining*** | | | |
| --- | --- | --- | --- |
| Antibody | Dilution | Catalog # | Company |
| Ki67 | Prediluted | clone 30-9 | Ventana BenchMark,  Basel, Switzerland |
| ***Immunoblots-primary antibodies*** | | | |
| Antibody | Dilution | Catalog # | Company |
| P(S616)-DRP1 | 1:1000 | 3455 | Cell Signaling,  Beverly, MA |
| MFN2 | 1:1000 | 9482 |  |
| ATF4 | 1:1000 | 11815 |  |
| PHGDH | 1:1000 | 13428 |  |
| ERK | 1:1000 | 9102 |  |
| P(T202/Y204)-ERK | 1:1000 | 9101 |  |
| AKT | 1:1000 | 4691 |  |
| P(S473)-AKT | 1:1000 | 4060 |  |
| CDK1 | 1:1000 | 9116 |  |
| CDK2 | 1:1000 | 2546 |  |
| CDK5 | 1:1000 | 14145 |  |
| MFN1 | 1:1000 | 13798-1-AP | Proteintech,  Rosemount, IL |
| OPA1 | 1:1000 | GTX48589 | GeneTex,  Hsinchu, Taiwan |
| DRP1 | 1:1000 | 611112 | BD Biosciences,  San Jose, CA |
| Total OXPHOS | 1:400 | ab110411 | Abcam,  Cambridge, MA |
| TOM20 | 1:500 | sc11415 | Santa Cruz,  Dallas, Texas |
| β-Actin | 1:5000 | MAB1501 | Millipore,  Burlington, MA |
| ***Immunoblots-secondary antibodies*** | | | |
| Antibody | Dilution | Catalog # | Company |
| Anti-rabbit IgG | 1:5000 | 213110 | GeneTex,  Hsinchu, Taiwan |
| Anti-mouse IgG | 1:5000 | 405306 | BioLegend,  San Diego, CA |
