# Supplementary material for: Multi‐kinase framework promotes proliferation and invasion of lung adenocarcinoma through activation of dynamin‐related protein 1
Source: Mol Oncol. 2020 Dec 11;15(2):560–78. doi: 10.1002/1878-0261.12843 (PMC7858280; doi:10.1002/1878-0261.12843)
Supplement: Supplementary file 3 — Table S2. Nucleotide sequences for qPCR primer pairs and guide RNA for CRISPR/Cas9 knockout. [file MOL2-15-560-s003.docx]

**Table S2**. Nucleotide sequences for qPCR primer pairs and guide RNA for CRISPR/Cas9 knockout

| **Gene** | **Nucleotide sequences** |
| --- | --- |
| *qPCR primer pairs for RNA expression* | |
| *PCK2* [1] | F: 5’-CATCCGAAAGCT CCCCAAGT-3’  R: 5’-GCTCTCTACTCG TGCCACAT-3’ |
| *PHGDH* [1] | F: 5’-GCAAAGAGGAGCTGATAGCG-3’  R: 5’-TTCTCAGCTGCGTTGATGAC-3’ |
| *PSAT1* | F: 5’-ACTTCCTGTCCAAGCCAGTGGA-3’  R: 5’-CTGCACCTTGTATTCCAGGACC-3’ |
| *PSPH* [1] | F: 5’-GAGGACGCGGTGTCAGAAAT-3’  R: 5’-GGTTGCTCTGCTATGAGTCTCT-3’ |
| *TBP* [2] | F: 5’-CACGAACCACGGCACTGATT-3’  R: 5’-TTTTCTTGCTGCCAGTCTGGAC-3’ |
| *qPCR primer pairs for mtDNA quantification* | |
| *ND1* [3] | F: 5’-ATACCCATGGCCAACCTCCT-3’  R: 5’-GGGCCTTTGCGTAGTTGTAT-3’ |
| *B2M* [4] | F: 5’-TGCTGTCTCCATGTTTGATGTATCT-3’  R: 5’-TCTCTGCTCCCCACCTCTAAGT-3’ |
| *gRNA for CRISPR/Cas9 gene knockout* [5] | |
| *DNM1L* | 5’- GCTGCCTCAAATCGTCGTAG-3’ |
| *CDK2* | 5’- TCCCCAGAGTCCGAAAGATC-3’ |

**References**

1. Quiros PM, Prado MA, Zamboni N, D'Amico D, Williams RW, Finley D, Gygi SP, Auwerx J (2017) Multi-omics analysis identifies ATF4 as a key regulator of the mitochondrial stress response in mammals. *J Cell Biol* **216**, 2027-2045.

2. Bieche I, Onody P, Laurendeau I, Olivi M, Vidaud D, Lidereau R, Vidaud M (1999) Real-time reverse transcription-PCR assay for future management of ERBB2-based clinical applications. *Clin Chem* **45**, 1148-1156.

3. Nakahira K, Kyung SY, Rogers AJ, Gazourian L, Youn S, Massaro AF, Quintana C, Osorio JC, Wang Z, Zhao Y *et al.* (2013) Circulating mitochondrial DNA in patients in the ICU as a marker of mortality: derivation and validation. *PLoS Med* **10**, e1001577.

4. Venegas V, Halberg MC (2012) Measurement of mitochondrial DNA copy number. *Methods Mol Biol* **837**, 327-335.

5. Sanjana NE, Shalem O, Zhang F (2014) Improved vectors and genome-wide libraries for CRISPR screening. *Nat Methods* **11**, 783-784.
