# Supplementary material for: Multi‐kinase framework promotes proliferation and invasion of lung adenocarcinoma through activation of dynamin‐related protein 1
Source: Mol Oncol. 2020 Dec 11;15(2):560–78. doi: 10.1002/1878-0261.12843 (PMC7858280; doi:10.1002/1878-0261.12843)
Supplement: Supplementary file 4 — Table S3. Clinical characteristics of the study population. [file MOL2-15-560-s004.docx]

| **Table S3**. Clinical characteristics of the study population | | | |
| --- | --- | --- | --- |
| Parameters | Post-operative recurrence within 5 years | | *p* |
|  | No  (n=162) | Yes  (n=49) |  |
| Sex |  |  |  |
| Male | 61 (37.7) | 24 (49.0) | 0.157 |
| Female | 101 (62.3) | 25 (51.0) |  |
| Age | 58.7±11.6 | 63.9±10.9 | 0.006 |
| Stage |  |  |  |
| IA | 120 (74.1) | 11 (22.4) | <0.001 |
| IB | 28 (17.3) | 15 (30.6) |  |
| IIA | 3 (1.9) | 9 (18.4) |  |
| IIB | 3 (1.9) | 3 (6.1) |  |
| IIIA | 8 (4.9) | 11 (22.4) |  |
| Data is presented as mean±standard deviation or number (percentage). Continuous variables were compared by a Student’s t-tests and categorical variables by a Pearson’s chi-square test. A *p* value less than 0.05 was considered statistically significant. | | | |
