# Supplementary material for: Multi‐kinase framework promotes proliferation and invasion of lung adenocarcinoma through activation of dynamin‐related protein 1
Source: Mol Oncol. 2020 Dec 11;15(2):560–78. doi: 10.1002/1878-0261.12843 (PMC7858280; doi:10.1002/1878-0261.12843)
Supplement: Supplementary file 5 — Table S4. Multi‐variate Cox proportional hazard ratio model for 5‐year post‐operative recurrence. [file MOL2-15-560-s005.docx]

| **Table S4**. Multi-variate Cox proportional hazard ratio model for 5-year post-operative recurrence | | | |
| --- | --- | --- | --- |
| Covariates | Hazard ratio | 95% Confidence interval | *p* |
| DRP1 H-score (high vs. low)^a^ | 2.451 | 1.266~4.744 | 0.008 |
| Age | 1.007 | 0.981~1.035 | 0.587 |
| Stage |  |  |  |
| IA | 1 | − | − |
| IIB | 3.810 | 1.666~8.709 | 0.002 |
| IIA | 9.836 | 3.893~24.853 | <0.001 |
| IIB | 9.719 | 2.545~37.123 | 0.001 |
| IIIA | 9.317 | 3.837~22.620 | <0.001 |
| P(S616)-DRP1 H-score (high vs. low)^a^ | 5.261 | 2.008~13.785 | 0.001 |
| Age | 0.993 | 0.966~1.020 | 0.594 |
| Stage |  |  |  |
| IA | 1 | − | − |
| IB | 3.518 | 1.559~7.938 | 0.002 |
| IIA | 9.279 | 3.640~23.655 | <0.001 |
| IIB | 8.150 | 2.118~31.358 | 0.002 |
| IIIA | 9.128 | 3.787~22.000 | <0.001 |

The hazard ratio of DRP1 or P(S616)-DRP1 H-score was calculated and adjusted using the parameters which were significantly associated with post-operative recurrence by univariate analyses in Table S3.

^a^The cutoff for dichotomization was determined by receiver operating characteristic curves and the Youden index.
